# Toward Next Generation Lateral Flow Assays: Integration of Nanomaterials

**DOI:** 10.1021/acs.chemrev.1c01012

**Published:** 2022-09-06

**Authors:** Amadeo Sena-Torralba, Ruslan Álvarez-Diduk, Claudio Parolo, Andrew Piper, Arben Merkoçi

**Affiliations:** † Nanobioelectronics & Biosensors Group, Institut Català de Nanociència I Nanotecnologia (ICN2), CSIC and The Barcelona Institute of Science and Technology (BIST), Campus UAB, Bellaterra, 08193 Barcelona, Spain; ‡ Instituto Interuniversitario de Investigación de Reconocimiento Molecular y Desarrollo Tecnológico (IDM), Universitat Politècnica de València, Universitat de València, Camino de Vera s/n, 46022 Valencia, Spain; § Barcelona Institute for Global Health (ISGlobal) Hospital Clínic−Universitat de Barcelona, Carrer del Rosselló 132, 08036 Barcelona, Spain; ∥ Catalan Institution for Research and Advanced Studies (ICREA), Pg. Lluís Companys 23, 08010 Barcelona, Spain

## Abstract

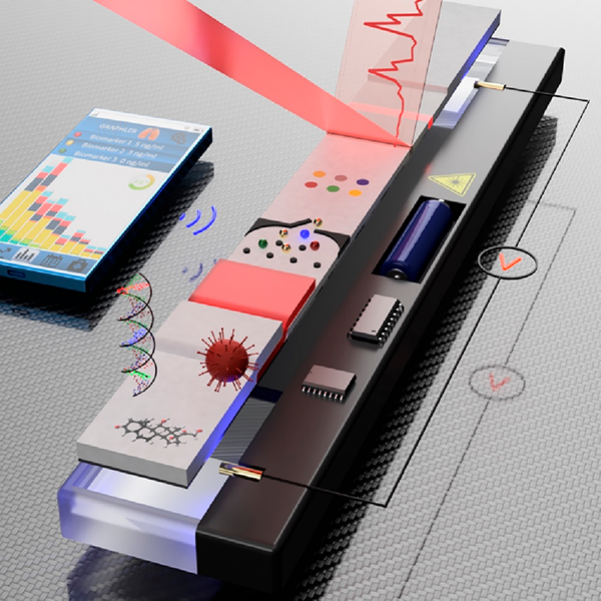

Lateral flow assays (LFAs) are currently the most used point-of-care sensors for both diagnostic (e.g., pregnancy test, COVID-19 monitoring) and environmental (e.g., pesticides and bacterial monitoring) applications. Although the core of LFA technology was developed several decades ago, in recent years the integration of novel nanomaterials as signal transducers or receptor immobilization platforms has brought improved analytical capabilities. In this Review, we present how nanomaterial-based LFAs can address the inherent challenges of point-of-care (PoC) diagnostics such as sensitivity enhancement, lowering of detection limits, multiplexing, and quantification of analytes in complex samples. Specifically, we highlight the strategies that can synergistically solve the limitations of current LFAs and that have proven commercial feasibility. Finally, we discuss the barriers toward commercialization and the next generation of LFAs.

## 1 Introduction

In 2003, the World Health Organization (WHO) published its ASSURED criteria which outlined the ideal test for universally available diagnostics. These criteria stipulate that point-of-care (PoC) diagnostics should be Affordable, Sensitive, Specific, User-friendly, Rapid and robust, Equipment-free, and Deliverable to end-users. Two additional criteria, R (real-time connectivity) and E (ease of specimen collection and environmental friendliness), were added then to the original ASSURED, creating the new acronym of REASSURED.^1^ Various PoC diagnostic platforms have been developed to realize biosensors that fulfill these criteria in the nearly two decades since they were published. Perhaps none more so than lateral flow assays (LFAs), which have risen to prominence thanks to their low cost and ease of use.^2,3^ LFAs are currently the most used PoC sensors for diagnostic^4^ (e.g., pregnancy test,^5^ COVID-19 monitoring^6,7^), environmental (e.g., pesticides,^8^ heavy metals,^9^ and bacterial monitoring^10^), and food safety applications (e.g., foodborne allergens^11^ and pathogens^12^).^13^ The main reason that LFAs are so popular is because they are made on paper-based substrates. Paper-based materials enable low-cost and sustainable manufacturing, while their porous matrices provide equipment-free microfluidics (driven by capillary forces), a biocompatible scaffold capable of supporting biointeractions (e.g., between antibodies and antigens), and a flexible substrate on which diverse nano-biosensing designs and strategies can be developed (e.g., origami, barriers, and constrictions).^14^ The fundamentals of LFA have been extensively covered in several peer-reviewed manuscripts and guides.^15,16^

While LFAs are extremely versatile, they are still limited to the qualitative detection of a single highly concentrated analyte, lacking the sensitivity required for low-concentration analyte detection, especially in complex biological matrices. As such, much of the recent research on nanoparticle-based LFAs has focused on increasing their sensitivity,^17−19^ multiplexing,^20,21^ and novel methods to allow measurements in complex media,^22,23^ as well as for detecting new biomarkers. However, most publications will seek to address each of these issues individually. To the best of our knowledge this is the first review that brings together the most recent, cutting-edge approaches obtained by the scientific community during the last 10 years, emphasizing the strategies that can synergistically solve the inherent challenges of developing PoC LFAs for clinical applications. This is vital in order to achieve the next generation of LFAs. The Review finishes by discussing the barriers toward commercialization and where lie the future perspectives of the field.

## 2 Quantification at the PoC

Quantitative analysis is defined as the ability to define the concentration of an analyte in a sample matrix.^24^ Its use in LFAs makes the assays much more powerful than the binary “yes/no” answer obtained from qualitative LFAs.^25^ For some applications, such as pregnancy tests, a simple binary readout is sufficient; the person taking the test is either pregnant or not. However, assay quantification is vital in applications where the concentration at which the target analyte is present can be used to inform decision making or where changes in concentration need to be monitored. Such examples include tests for analytes that are present under normal conditions but whose concentrations can be elevated or decreased in certain conditions. Perhaps the best example of this is a glucose sensor: a binary test saying that glucose is present in a sample matrix is not desirable; however, a quantifiable assay that determines whether the glucose concentration in a sample is higher or lower than accepted healthy limits is. There are a multitude of more complex clinical scenarios (e.g., cancer), where variations in the levels and ratios of several biomarkers (molecule’s overexpressions and downregulations) give insight on the efficacy of therapies and disease progression/prognosis.^26,27^ Similar scenarios exist in food and environmental safety applications, where, for instance, the slight increase of food-borne allergenic content or pollutant concentrations in a river beyond accepted safe limits can have lethal consequences.^11,28^

The potential to quantify the signal on an LFA is determined by the nature of the signal generated at the test line (TL) which needs to be in some way related to the concentration of biomarker in the sample. The signal obtained must then be compared to a calibration curve obtained by performing the assay with known concentrations of the analyte across the relevant concentration range in as similar a sample matrix as possible.^29^

Quantification in LFAs has been performed by purpose-built readers with simple operation procedures. In all cases these should be designed to be portable, robust, and cheap for on-site/PoC use. Recent advances have included the development of software and apps as well as readers for more complex signal analysis, including using new techniques (e.g., SERS) and novel LFA design architectures (e.g., microarrays).

In this section, we will review the recent advances in the development of LFA readers capable of LFA quantification in PoC/on-site scenarios. We compare the operation mode, price, dimensions, and reported assay sensitivity for each reader (Table 1) and will give greater emphasis to those that are best suited to commercialization.

**Table 1:** Comparison of the Readout Systems for LFA Quantification

reader	signal	detection of	LoD (pg mL^–1^)	price ($)	size (cm^3^)	weight (g)	ref
smartphone-based	colorimetric	*E. coli* O157:H7	10^4^ CFU mL^–1^	∼200	2.5 × 3.8 × 2	400	(38)
smartphone-based	colorimetric	HIgG	1.00 × 10^04^	∼250	1.5 × 0.8 × 0.1	140	(39)
smartphone-based	colorimetric	EVD-IgG	2.00 × 10^05^	∼200	13.6 × 6.9 × 0.7	130	(4)
smartphone-based	colorimetric	HIgG	7.50 × 10^04^	214	14 × 7 × 1	130	(40)
smartphone-based	luminescence	PSA	100	∼200			(41)
smartphone-based	colorimetric and luminescence	AFB1	590	∼200	7 × 7 × 7		(42)
UCNP reader	luminescence	triamcinolone acetonide	980		30 × 30 × 15	2000	(47)
UCNP reader	luminescence	ST-2	5000	∼700	24 × 9.4 × 5.4	900	(48)
UCNP reader	luminescence	ochratoxin A	3000	∼650			(49)
thermal contrast reader	TCA	hCG	180	∼500	13.3 × 10.8 × 7.3		(58)
SERS reader	SERS	hCG	106	>3000	18 × 11 × 4.7	>1000	(59)
photoacoustic reader	PA	glucose	5.40 × 10^06^	∼1300	4 × 2 × 2	>4000	(60)

### 2.1 LFA Semiquantification by Naked Eye

Semiquantification relies on determining an approximate quantity of the target analyte by visual inspection, without the need for external instrumentation. The methods to make an LFA semiquantifiable are to (i) relate a particular signal intensity or color tonality with a target concentration,^30^ (ii) design the assay so that the signal appears only when the detected target analyte is above a threshold value,^31^ or (iii) represent different target analyte concentrations using different test lines.^32^ However, Saiykhan et al. have developed an innovative method that enables the semiquantification of blood plasma fibrinogen, based on the flow rate and distance traveled by the sample. In this work a wax-printed chromatography paper strip was functionalized with bovine thrombin. This causes the soluble fibrinogen to precipitate as the sample flows along the strip. Thus, the higher the concentration of fibrinogen in the sample, the lower the flow rate and distance traveled. The paper strip is placed inside a holder with a built-in scale (with millimeter resolution). This enables the visual monitoring of the distance traveled by the sample. The device is simple, portable, and cost-effective, enabling the PoC semiquantification of fibrinogen in a range of 0.5 to 7.0 mg mL^–1^.^33^ The use of rulers has also been reported by Li et al., albeit in a much more complex system. The working principle of their assay relies on the use of antibody-functionalized platinum nanoparticles (PtNPs) that are captured on the TL as in a conventional LFA. After the assay has run, the TL is cut out of the strip and inserted into a sample reservoir at the base of a microfluidic ruler. The PtNPs catalyze the generation of oxygen that pushes red ink along the microfluidic channel. The distance traveled by the ink within a specific time can be directly related to the concentration of PtNPs captured on the TL and, therefore, to the concentration of target analyte present in the sample. The authors have applied this approach for the detection of prostate specific antigen (PSA) in clinical samples, achieving a limit of detection (LoD) of 0.54 ng mL^–1^ and a linear range of 0–12 ng mL^–1^.^34^

### 2.2 Smartphone-Based Readers

Rather than developing new hardware for LFA quantification and analysis, it would be beneficial to use ubiquitously available technologies for assay analysis. Smartphones have revolutionized the way we live; their portability, accessibility, widespread use, and sophistication have motivated their use as LFA readout systems for PoC quantification.^35^ Modern smartphones are an interesting alternative to conventional benchtop readers, since they are portable miniaturized computers with large random access memory, high-speed CPUs, sophisticated camera lenses with Wi-Fi/Bluetooth, and IR network connectivity.^36^ Thus, they possess all the features required of a PoC/on-site device: capable of assay quantification and data storage and transfer.^37,36^ When taking pictures of an LFA for quantification, it is important that the lighting conditions and optical settings of the camera are optimized and kept constant. Variations in these factors can cause variations in the measured biomarker concentration. In this section, we will review recent advances in the use of smartphones for LFA quantification.

**Figure 1:**
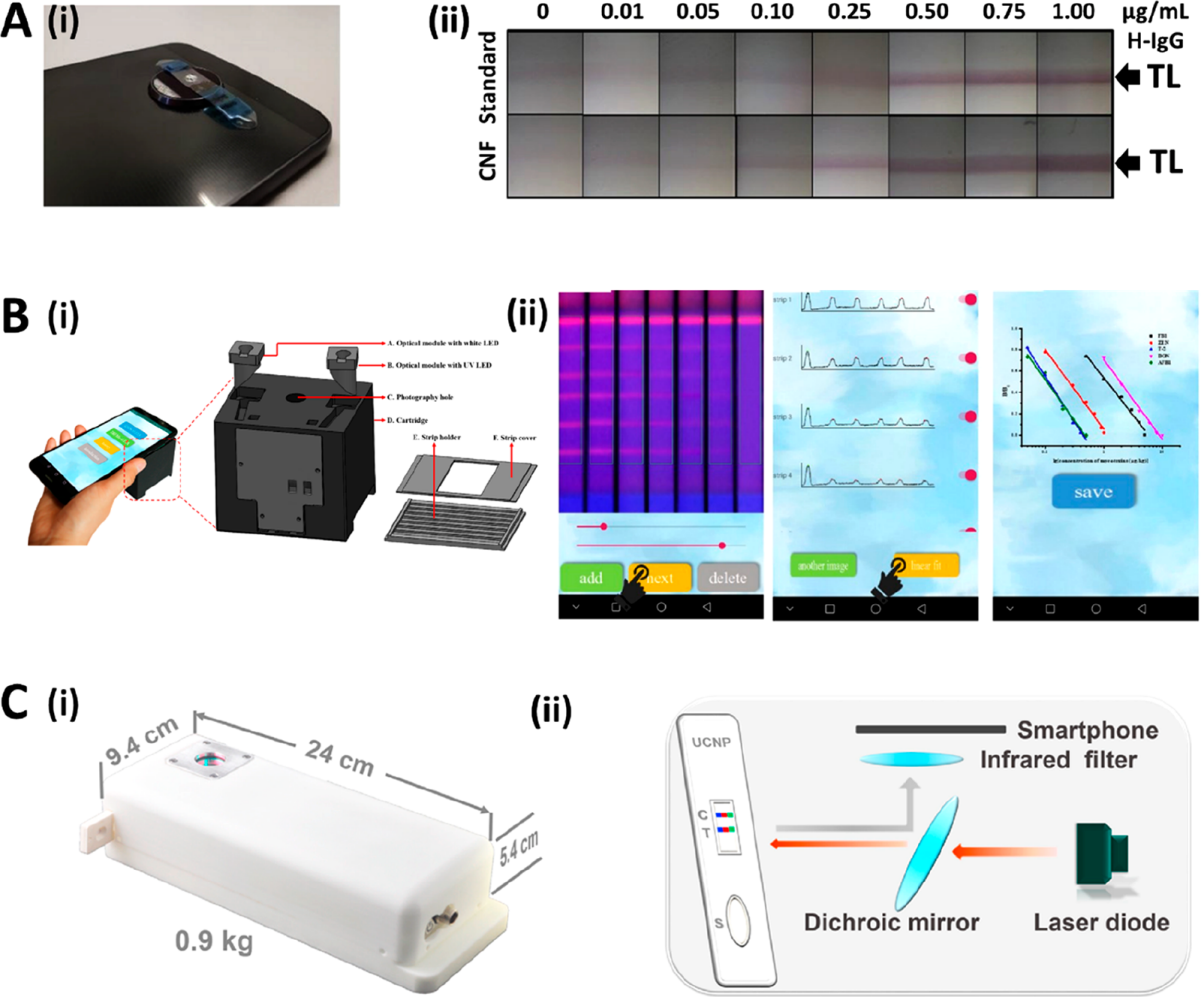
(A, i) Picture of microscope lenses attached to a smartphone camera that were used to photograph the detection area colorimetric LFA strips. (A, ii) Picture of the LFA strips taken with the microscopic lens and the smartphone camera. Reprinted (adapted) with permission from ref (39). Copyright 2019 Elsevier. (B, i) Schematic representation of a 3D-printed cartridge that contains all the optical apparatus for smartphone-based quantification of colorimetric and fluorescent LFAs. (B, ii) Screenshots from the universal detection app, showing the image analysis features for assay quantification. Reprinted (adapted) with permission from ref (42). Copyright 2020 Elsevier. (C, i) Picture of a portable UCNPs-LFA reader with dimensions of 24 × 9.4 × 5.4 cm and 0.9 kg weight. (C, ii) Schematic representation of the evaluation of the strips, in which the laser light is transmitted by the dichroic mirror at 45° to the detection zone. The smartphone camera captures the luminescence emission of the UCNPs present in the test and control lines, while the infrared filter blocks the laser’s residual light. Reprinted (adapted) with permission from ref (48). Copyright 2019 Elsevier.

The use of 3D printers allows the fabrication of mobile phone supports that guarantee the correct lighting and distance between the sample and the camera. This enables the reproducible collection of images for quantification.^15^ For example, Jung et al. have developed a smartphone-based reader consisting of a 3D-printed holder, a smartphone, and a preinstalled dedicated android app. The holder includes a smartphone cradle with optical lens that provides a 3× image amplification and an LED light source with a reflector that focuses the light into a diffuser that spreads the light evenly across the strip. Moreover, the android app incorporates data analysis software that calculates the concentration of analyte in the sample based on a prestored calibration curve. The authors report an LoD of 10^4^ CFU/mL for the direct detection of *Escherichia coli* O157:H7 in ground beef.^38^ In another work, Quesada et al. improved the performance of their smartphone-based readout by attaching a cheap (20 USD) and commercially available microscopic lens onto their smartphone camera (Figure 1Ai). This increases the number of pixels that can be recorded, improving the quality of high-magnification images taken of the test area (Figure 1Aii).^39^

Other researchers have exploited other smartphone capabilities. For instance, Brangel et al. have taken advantage of the smartphone’s GPS technology to develop an app that enables the geotagging of the samples to aid. They proved the applicability of this feature for Ebola surveillance screening and management of infected patients in Uganda.^4^ Miller et al. have used smartphone video analysis as a cost-effective and simple method to calculate the dissociation constant (*K*_D_) of analyte and bioreceptor in an LFA. The authors suggest that this strategy can overcome several barriers to the quantitative analysis of LFAs, such as color inhomogeneity and reproducibility. The achieved *K*_D_ showed excellent agreement with a reference benchtop interferometer.^40^

Smartphone-based readers have also been applied for the quantification of fluorescent LFAs. Danthanarayana et al. have developed a portable imaging instrument for the evaluation of nanophosphor-based LFAs. In this case, the authors used the flash of the smartphone camera to excite the luminescent nanoparticles for 3 s; then, an image is acquired after a 100 ms delay. This time-gated imaging is a commonly used strategy to decrease the background signal since it allows the applied light to decay before the image is acquired. This approach enabled the detection of PSA and hCG with LoDs of 0.1 and 1 ng/mL, respectively.^41^ Liu et al. have developed a smartphone-based reader for the dual quantification of colorimetric and fluorescent LFAs. They 3D-printed a 70 mm long cubic box using black photosensitive materials. The cartridge contains both white and UV LEDs as light sources for visible and fluorescent signaler excitation (Figure 1Bi). The images taken with the smartphone camera were analyzed with an Android Studio app that splits the image into red, green, and blue channels, with each channel being used to detect a different signaler in a multiplexed LFA (Figure 1Bii). The results obtained showed good consistency when validated against a liquid chromatography mass spectrometer (LC-MS).^42^

Upconverting nanoparticles (UCNPs) are luminescent nanomaterials that display anti-Stokes shifts; i.e., the emitted photon has a higher energy than the incident photon. In such circumstances, it is possible to irradiate with light in the NIR range of the spectrum and measure light emitted in the visible or ultraviolet range.^43^ UCNPs have several physical properties that make them suited for LFA quantification such as narrow emission spectra, high chemical stability, long luminescence lifetimes, and high resistance to photoquenching and photobleaching.^44^ The evolution of the UCNP reading systems has gone from the use of bulky microtiter plate readers^45^ to the use of custom-built commercially available fluorescent readers (UCP-Quant)^46,47^ and, in recent years, the development of smartphone-based readers. Gong et al. have developed a compact (24 × 9.4 × 5.4 cm) and ultralight (0.9 kg) static image acquisition device (Figure 1Ci). The device consists of a compact infrared laser (exc. at 980 nm), a dichroic mirror (with 98% light transmission at 980 nm), and an infrared filter with nearly 0% transmittance at 980 nm (Figure 1Cii). The image is acquired with a smartphone with an image analysis app that enables simple user operation and easy result interpretation. The device has been validated for the detection of nucleic acids, small proteins, heavy metal ions, and bacteria, making this UCNPs-LFA reader ideally suited for commercialization.^48^

Similarly, Jin et al. have developed a portable detection reader based on a 980 nm laser that excites the TL at a 45° angle. They have evaluated UCNP-LFA strips with a CCD camera (Nikon) and a smartphone (and using ImageJ software). They found that the smartphone was not as sensitive as the camera but still had a good linear correlation for ochratoxin A, mercury ions, and salmonella.^49^ This result is not surprising and highlights the importance of the camera/smartphone in these readers, which is often overlooked in publications.

### 2.3 Portable LFA Readers for Alternative Signal Transduction Methods

Optical detection methods using colorimetric or fluorescent labels to give a visual readout are by far the most popular type of LFAs. However, despite their simplicity, cost-effectiveness, and fast acquisition times, these detection methods typically suffer from poor sensitivities, high noise levels, and low reproducibility.^50^ As mentioned previously, this has led to the development of alternative signal transduction methods; however, these alternative methods need portable readers for PoC/on-site assay quantification. In this section, we will review advances in PoC readers for other (nonvisual) transduction methods

#### 2.3.1 Magnetic Readers

Magnetic nanoparticles have been extensively utilized in LFAs for sample pretreatment, particularly for the isolation and preconcentration of the analyte. However, these nanoparticles also offer outstanding analytical capabilities when used as labels in magnetometric detection.^51^ There are currently two reported ways of quantifying magnetic nanoparticles, by inductive sensing and by magnetic particle quantification (MPQ). Both require a coil that excites the magnetic nanoparticles and interrogates the magnetic signal generated at the TL of the LFA strip. The inductive sensing measures the magnitude and phase of the coil impedance, which varies depending on the change in magnetic permeability of the magnetic nanoparticles.^52^ In MPQ the coil generates a nonlinear magnetic field that alternates between two frequencies, *f*_1_ and *f*_2_. The signal emitted by the nanoparticles after their magnetization is recorded by a coil attached to a magnetic reader.^53^

**Figure 2:**
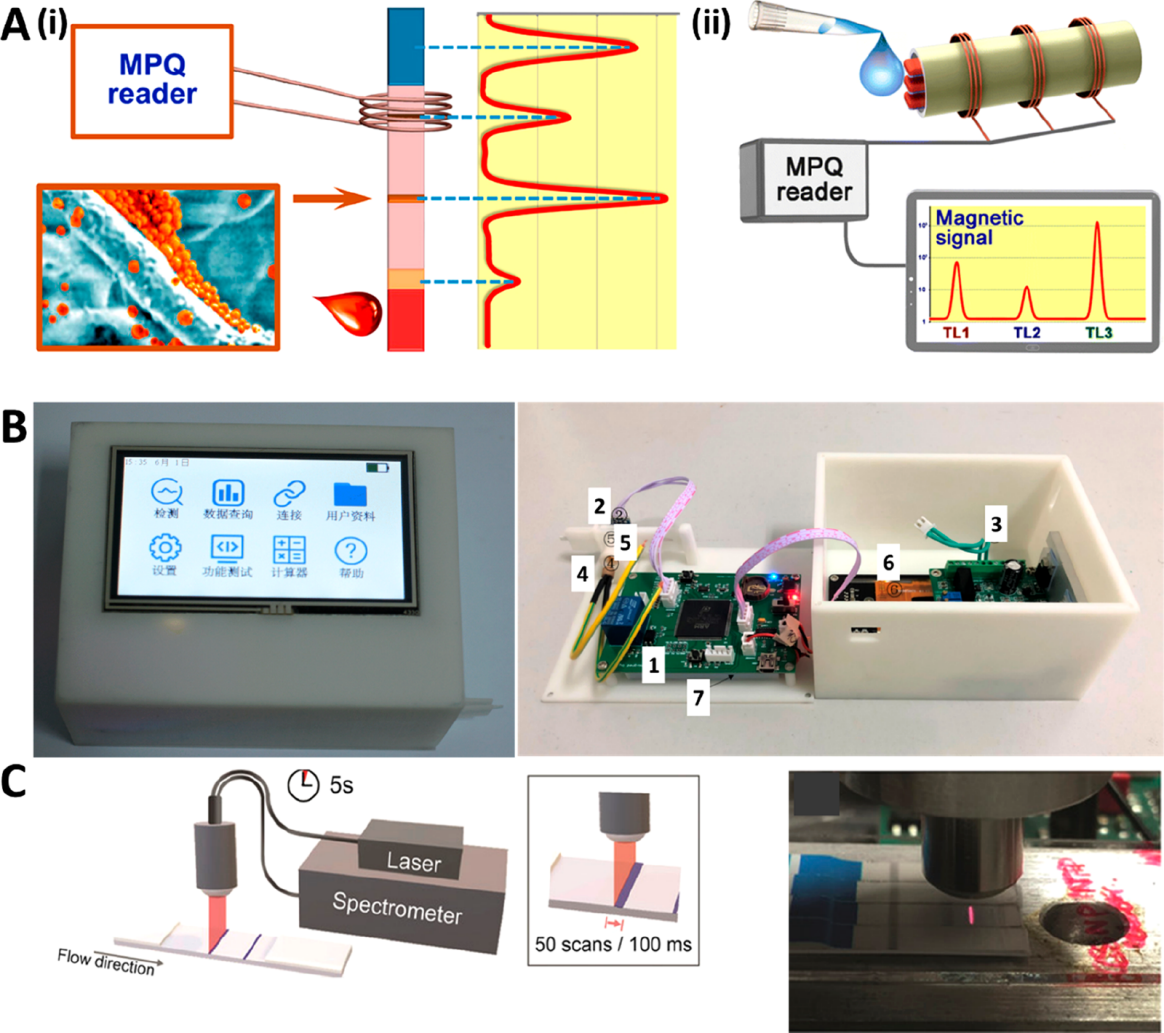
(A, i) Schematic representation of the MPQ quantification procedure of LFAs using a PoC MPQ reader. Reprinted (adapted) with permission from ref (54). Copyright 2016 Elsevier. (A, ii) Schematic representation of the simultaneous quantification of three LFA strips using a cartridge and the MPQ reader. Reprinted (adapted) with permission from ref (51). Copyright 2016 American Chemical Society. (B) Pictures of a portable LFA reader used for the quantification of the heat generated on the LFA TL due to thermal contrast amplification. The reader includes sensors for the evaluation of both heat conduction and radiation. Reprinted (adapted) with permission from ref (58). Copyright 2016 Springer. (C, i) Schematic representation of a SERS reader. (C, ii) Picture of the SERS reader during the scanning of the LFA strips. Reprinted (adapted) with permission from ref (59). Copyright 2019 Wiley.

Magnetic quantification offers several advantages over the traditional colorimetric reading, such as deep signal registration (it measures the signal generated in the TL volume rather than what is observable at the NC surface), extremely wide dynamic linear ranges (up to 7 orders of magnitude), and better sensitivity when evaluating complex samples (the opacity of the biological sample does not hinder the magnetic readout). Based on these analytical features, some researchers have focused on developing portable and cost-effective readers capable of performing MPQ at the PoC. Orlov et al. have designed an MPQ method and portable reader with outstanding analytical properties. Their assay used 200 nm diameter magneto-radioactive ^59^Fe-based MPs conjugated to monoclonal anti-PSA. The assay quantification is performed by the incorporation of the test strip in the measuring coil of the MPQ reader (Figure 2Ai). They report an LoD of 25 pg mL^–1^ of PSA in human serum, a dynamic range spanning 3.5 orders of magnitude, and a high dose–response sensitivity of *k*_log_ = 3. The authors also suggest that the developed MPQ reader can perform real-time MP mapping during the biorecognition event. This could be a useful tool for the rapid determination of antibody–antigen binding kinetics.^54^ The MPQ reader is also suited to multiplexing strategies, by the use of several coils that enable the interrogation of different TLs present on a single strip^55^ or by the simultaneous evaluation of several strips in a cartridge (Figure 2Aii).^56^ MPQ readers are still in the early development stage with most prototypes only existing in research laboratories, and none are commercially available at the time of writing. This makes it difficult to assess the commercial viability of such devices.

#### 2.3.2 Thermal Contrast Readers

The use of TCA has been widely reported in recent years as a strategy to improve the sensitivity of LFAs. As previously explained in section 2, the working principle relies on the light-to-heat transduction of metallic nanoparticles upon their irradiation with an NIR laser. The cornerstone of this sensing method is the high resolution of infrared cameras, which can recognize temperature differences of just 0.1 °C, providing high assay sensitivity compared to image analysis of optical readout methods. As was the case with magnetic detection, TCA can overcome the light scattering issues of colorimetric detection systems. Recent research has focused on the development of portable alternatives to the current benchtop TCA readers.^57^ Qu et al. have developed the first portable thermal contrast reader. It is small (133 × 108 × 73 mm) and has been fabricated with low-cost components (Figure 2B). They validate their reader with an LFA that uses anti-hCG functionalized Au nanoprisms. Once the LFA strip is placed in the reader, an NIR light beam is focused on the TL. The gold nanoprisms absorb the light and generate heat, which is detected by either a semiconductor sensor (heat conduction sensing) or infrared thermometer (heat radiation sensing). The semiconductor sensor has a resolution of 16 bit (0.0078 °C), while the infrared thermometer has a slightly higher 17 bit resolution (0.0039 °C). the higher sensitivity of the radiation method did however make it more prone to noise. The researchers recommend the selection of either sensing mode depending on the assay requirements. This approach yielded a 12-fold lower LoD (180 pg mL^–1^) than the comparable AuNP-based LFA for hCG detection.^58^

#### 2.3.3 Surface-Enhanced Raman Scattering Readers

There have been several attempts to develop PoC SERS instruments. For instance, Tran et al. have developed a portable SERS-LFA reader with a fiber optic probe able to acquire 50 scans at different positions on the TL in 5 s. In this work the strip was scanned orthogonally to the TL, and the motorized stage would move one step every 10 ms (Figure 2C). This realized a rapid test with data acquisition times several orders of magnitude shorter than conventional Raman microscopes, ideal for PoC applications. Moreover, the method was 15 times more sensitive than the conventional AuNP-based colorimetric detection of hCG (LoD of 0.16 ng mL^–1^).^59^

The main drawback to SERS readers being used in PoC applications is their cost. The most expensive components are the fiber optic Raman probes (>$2000) and the portable spectrometers (>$800). As well as finding ways to reduce the costs of these components, more powerful and user-friendly software will also need to be developed before SERS readout platforms can be used in PoC LFA quantification applications.

## 3 Sensitivity Enhancement

As for any diagnostic device, the optimization and design of an LFA should be tailored toward the intended final use; for example, it needs to be sensitive across the clinically relevant biomarker range.^61^ Perhaps the most important analytical properties are sensitivity (i.e., the ability to discriminate between different analyte concentrations) and limit of detection (i.e., the lowest analyte concentration that can be discriminated from the blank).^62^ The vast majority of clinical applications require the detection of sub-picomolar concentrations of biomarkers. In this section, we present recent advances aimed at improving the LFA sensitivity when using the noncompetitive assay format (Table 2); we discuss their pros and cons as well as their commercialization feasibility.

**Table 2:** Comparison of the Sensitivity Enhancement Strategies Reported for LFA

strategy	method/material	detection of	enhancement	comp. to	ref
flow rate decrease	hydrophobic PCL nanofibers	synthetic Zika virus	10-fold	conv. LFA	(65)
flow rate decrease	cellulose nanofiber aerogel	mouse IgG	1000-fold	conv. LFA	(66)
flow rate decrease and reagents concentration	reduce detection area from 5 mm to 1 mm	C-reactive protein	30-fold	conv. LFA	(67)
flow rate decrease and pseudoturbulence generation	wax pillars	HIgG	3-fold	conv. LFA	(69)
internal incubation on TL	dissolvable wax barrier	HIgG	51.7-fold	conv. LFA	(70)
flow control	centrifugal forces	PSA	6.2-fold	conv. LFA	(72)
sample preconcentration	magnetic focusing	valosin-containing protein	4000-fold	no preconc.	(73)
sample preconcentration	magnetic focusing	influenza A (H1N1) antigen	26-fold	no magnetic focusing	(74)
sample preconcentration	filtration	*E. coli*	10-fold	no filtration	(10)
sample preconcentration	Nafion-based ICP	β-hCG	3.9-fold	no preconc.	(75)
increase antibodies concentration	fixing conj. AuNPs in TL	*C. sakazakii*	100-fold	conv. LFA	(76)
increase signal contrast	carbon nanoparticles	*E. Coli*	3.8-fold	conv. LFA	(77)
increase signal contrast	MWCNTs	Meth.	10-fold	AuNPs-LFA	(78)
fluorescence signal transduction and sample pre-concentration	magnetic CdSe/ZnS QDs nanobeads	Influenza A	LoD of 22 pfu mL^−1^	no comparison	(81)
ratiometry with target-dependent and independent reporters	red CdSe/ZnS QDs and blue polystyrene nanobeads	H-IgG	78-fold	AuNPs-LFA	(82)
fluorescence signal transduction and increased signal-to-noise ratio	fluorescent nanodiamonds	biotin	10^5^-fold	AuNPs-LFA	(84)
signal amplification	silver staining of AuNPs	Troponin I	10-fold	AuNPs-LFA	(88)
signal amplification	AuNPs coated with HRP	HIgG	10-fold	AuNPs-LFA	(89)
signal amplification	AuNPs coated with ALP	potato virus X	10-fold	AuNPs-LFA	(90)
signal amplification	AuNPs coated with ultrathin Pt skins	PSA	100-fold	AuNPs-LFA	(91)
signal amplification	porous platinum core−shell nanocatalysts (PtNCs)	P24	100-fold	no catalysis	(92)
alternative signal transduction	thermal contrast amplification	influenza, malaria, and *C. difficile*	8-fold	AuNPs-LFA	(57)
alternative signal transduction	thermal contrast amplification	P24 protein	LoD of 8 pg mL^−1^	no comparison	(97)
alternative signal transduction	thermal contrast amplification	HCG	12-fold	AuNPs-LFA	(58)
alternative signal transduction	photoacoustic imaging	cryptococcal antigen (CrAg) from *Cryptococcus*	100-fold	AuNPs-LFA	(98)
alternative signal transduction and sample preconcentration	surface enhanced Raman scattering and magnetic focusing	H1N1 and HAdV viruses	2000-fold	AuNPs-LFA	(103)
alternative signal transduction and flow modulation	surface enhanced Raman scattering and sequential flow of Raman reporters	thyroid stimulating hormone (TSH)	LoD of 0.15 μIU/mL	no comparison	(104)
alternative signal transduction and bioreceptors	surface enhanced Raman scattering and bacteriophages as bioreceptors	*S. Enteritidis*	no enhancement	SERS-LFA based on antibodies	(105)

### ^3^.1 Modulation of the Flow Dynamics

One of the main reasons for the poor sensitivity in LFAs, when compared to other analytical techniques like enzyme-linked immunosorbent assays (ELISA), is that they do not allow as much time for the bioreceptors (probes) to bind to the antigens (targets).^63^ This is due to the capillary force driven wicking properties of the paper-based LFA strip, which produces a unidirectional flow of the sample at a constant rate through the substrate. Ideally the sample would be incubated on the test line (TL) to increase antigen capture, and therefore improve the sensitivity of the assay. There are two main methods of reducing the flow rate in LFAs: the first is by chemically modifying the nitrocellulose NC to reduce the flow rate, and the second is to change the geometry of the LFA components.

The earliest examples of chemical modification of LFAs to slow the flow rate involved the incorporation of salt barriers to impede the flow. These can be easily drop cast onto the nitrocellulose and will halt the flow until they have been dissolved by the sample solution. Usually these are placed after the test and control lines, but a recent work by Prof. Feng Xu and co-workers stands out because the salt barrier was placed in front of the test and control lines.^64^ Besides the reduction of the flow velocity and the consequent 10-fold sensitivity enhancement, the authors also claim that the increase in salt concentration improves the hybridization efficiency of the nucleic acid bioreceptors used. While this is a low-cost and simple approach, the high ionic strength of the sample solution at the target line will not be compatible with other bioreceptors and can denature some protein and aptameric receptors; therefore, its suitability should be evaluated for any given application.

**Figure 3:**
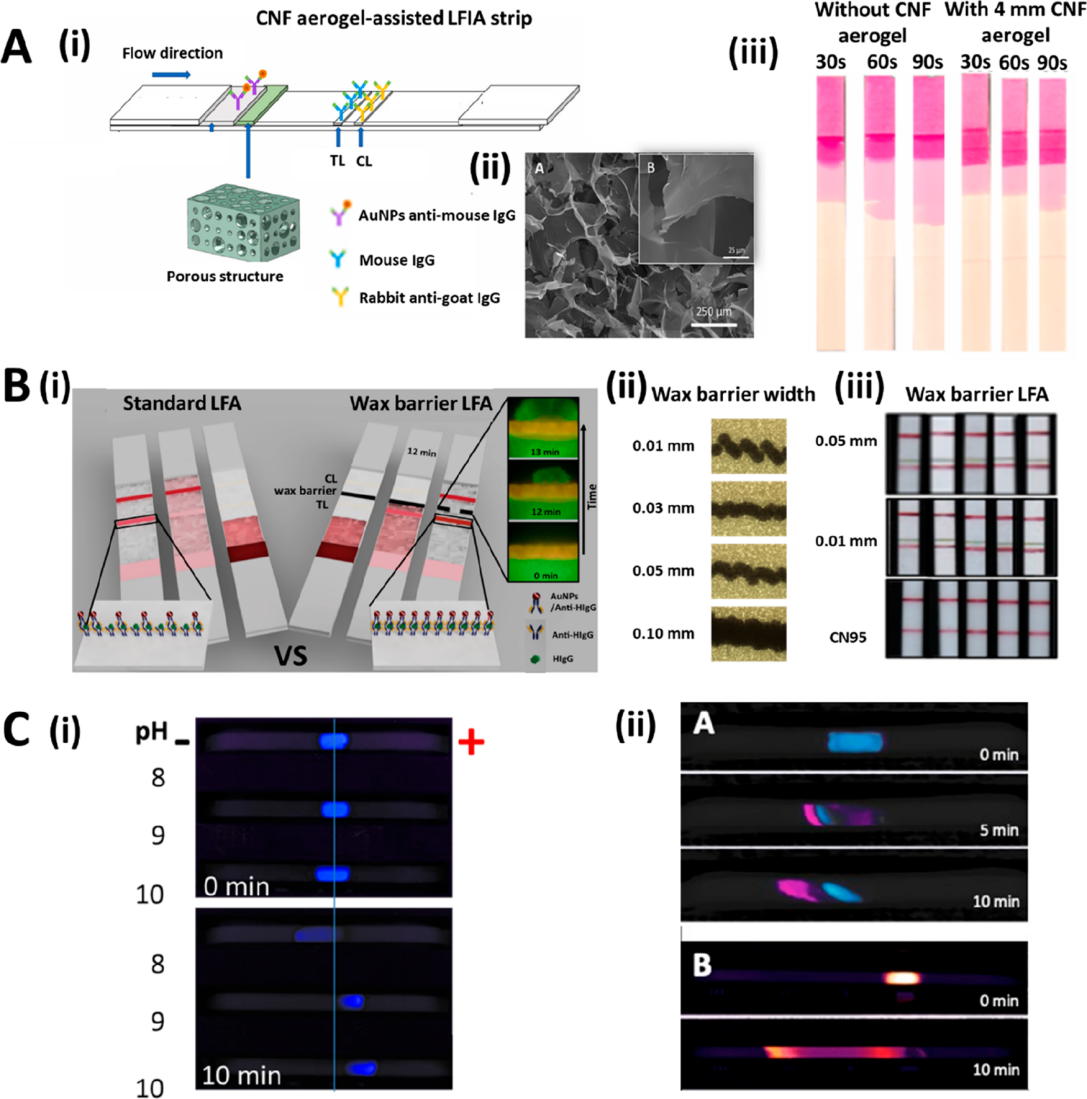
(A, i) Schematic representation of a sensitivity enhancement approach based on the introduction of a cellulose nanofiber (CNF) aerogel after the conjugate pad. (A, ii) SEM image of the CNF aerogel, showing an average pore size of 250 μm. (A, iii) Comparison of the flow after 30, 60, and 90 s in strips with and without the CNF aerogel. Reprinted (adapted) with permission from ref (66). Copyright 2021 Elsevier. (B, i) Schematic representation of a sensitivity enhancement approach based on the incorporation of a dissolvable wax barrier after the test line. (B, ii) Optical microscopy picture (40×) of wax barriers with different widths. (B, iii) Picture of the LFA strips with and without the wax barrier, after the detection of HIgG (0–1000 ng mL^–1^). Reprinted (adapted) with permission from ref (70). Copyright 2020 Elsevier. (C, i) Modulation of the direction of flow of graphene quantum dots by the adjustment of the electrolyte solution pH in a paper-based electrophoretic bioassay. (C, ii) Separation of a mixture of CdSe@ZnS QDs, CdTe QDs, and N,S-doped carbon dots according to the electrophoretic mobility of the nanomaterials. Reprinted (adapted) with permission from ref (71). Copyright 2021 American Chemical Society.

Recent advances in the field have focused on using polymeric chemical barriers as opposed to salts. For example, Yew et al. have electrospun a 10% solution of polycaprolactone nanofibers onto a nitrocellulose membrane for 60 s in order to delay the flow time by 17 s (0.29 mm/s). compared to uncoated nitrocellulose (NC) membranes (0.35 mm/s). The incorporation of polycaprolactone increases the hydrophobicity of the coated region, reducing the flow rate and increasing the interaction time between the biorecognition element and the analyte.^65^ This strategy resulted in a 10-fold increase in the sensitivity of the assay, compared to the conventional LFA. In such approaches it is important to consider the properties of the materials being incorporated into the strip, as these should in no way compromise the test performance. Tang et al. have shown that the insertion of a nanofibrillated cellulose aerogel (pore size of 100 nm in the wet state) just after the conjugate pad can decrease the sample flow rate in the lateral flow device (Figure 3A). The aerogel transforms into a hydrogel-like state upon wetting, with an almost 10^3^-fold pore size reduction compared to other reported stacking pads. This results in a 40–60% higher reaction time between the bioreceptors and the target analyte, which enables a 1000-fold sensitivity enhancement compared to the LFA without the aerogel (LoD of 0.72 ng mL^–1^ for mouse IgG in human serum). It is noteworthy that the aerogel has a shelf life of 6 months.^66^ Alternatively, Katis et al. have developed a strategy not based on the incorporation of novel materials, but on the reduction of the Test line width from 5 to 1 mm.^67^ They use a laser to polymerize a pattern on the nitrocellulose membrane. By narrowing the flow path, they achieve an increase in both the flow time and a concentration of the reagents. They report a 30-fold sensitivity enhancement for the detection of C-reactive protein compared to the assay with no flow focusing. The authors point out that this method is low-cost, since the polymerization can be performed using conventional lasers which is advantageous compared to other patterning techniques such as photolithography.^67^

Other works have also investigated changing the flow geometry to improve LFA sensitivity; for example, Parolo et al. have enlarged the width of the sample and conjugate pads 3-fold in order to increase the bed volume of the strip. The authors achieve an 8-fold enhancement in the limit of quantification (LoQ) for HIgG detection; the simplicity of this strategy makes it ideal for incorporation into other LFAs.^68^

Complex microfluidics can be cheaply, quickly, and easily introduced into LFAs, using wax printers, to improve the assay performance. For example, Rivas et al. introduced wax pillars at the beginning of the nitrocellulose membrane to produce a flow delay and generate pseudoturbulences in the capillary flow, both of which improve probe–analyte binding. Their method resulted in a 3-fold sensitivity enhancement for HIgG detection, in comparison to the conventional LFA.^69^ The position of the printed microfluidics along the nitrocellulose membrane affects the assay’s sensitivity. The incorporation of the wax pillars just after the conjugate pad (as in the case of Rivas et al.) enhances mainly the interaction time of the first biorecognition event, the binding of the target analyte, and the labeled bioreceptors, while the incorporation of such artifacts after the TL provides an increase in the interaction time of both the first and second biorecognition event, the second being the immuno-sandwich formation with the secondary antibody. By optimizing both binding events, Sena-Torralba et al. report a 51.7-fold sensitivity enhancement for HIgG detection by inserting a 0.05 mm wide dissolvable wax barrier 1 mm downstream of the TL (Figure 3B).^70^

A novel way of modulating the flow dynamics in LFAs is the application of alternative mobility forces, such as centrifugal or electrical forces. These provide the end-user with greater control of the flow rate and direction of the sample compared to capillarity, which is governed solely by the bed volume of the strip.^71^ To this end, Shen et al. report a centrifugal force-assisted LFA, with a constant rotation speed of 1500 rpm, adjusted by a portable dedicated device.^72^ This test enabled a 6.2-fold sensitivity enhancement for prostate specific antigen (PSA) detection in human serum.^72^ Likewise, Sena-Torralba et al. have developed an LFA that incorporates electrophoresis. In this way, the flow rate and direction of the sample can be precisely adjusted by the regulation of the voltage applied to, and pH of, the electrolyte solution (Figure 3Ci). The authors prove that this paper-based electrophoretic bioassay can separate a mixture of quantum dots of different sizes and charges in just 10 min, due to the differences in the electrophoretic mobility of both nanomaterials (Figure 3Cii).^71^ Based on this proof of concept, the researchers are currently working on the application of this approach for the betterment of the LFA sensitivity.

### 3.2 Sample Preconcentration

**Figure 4:**
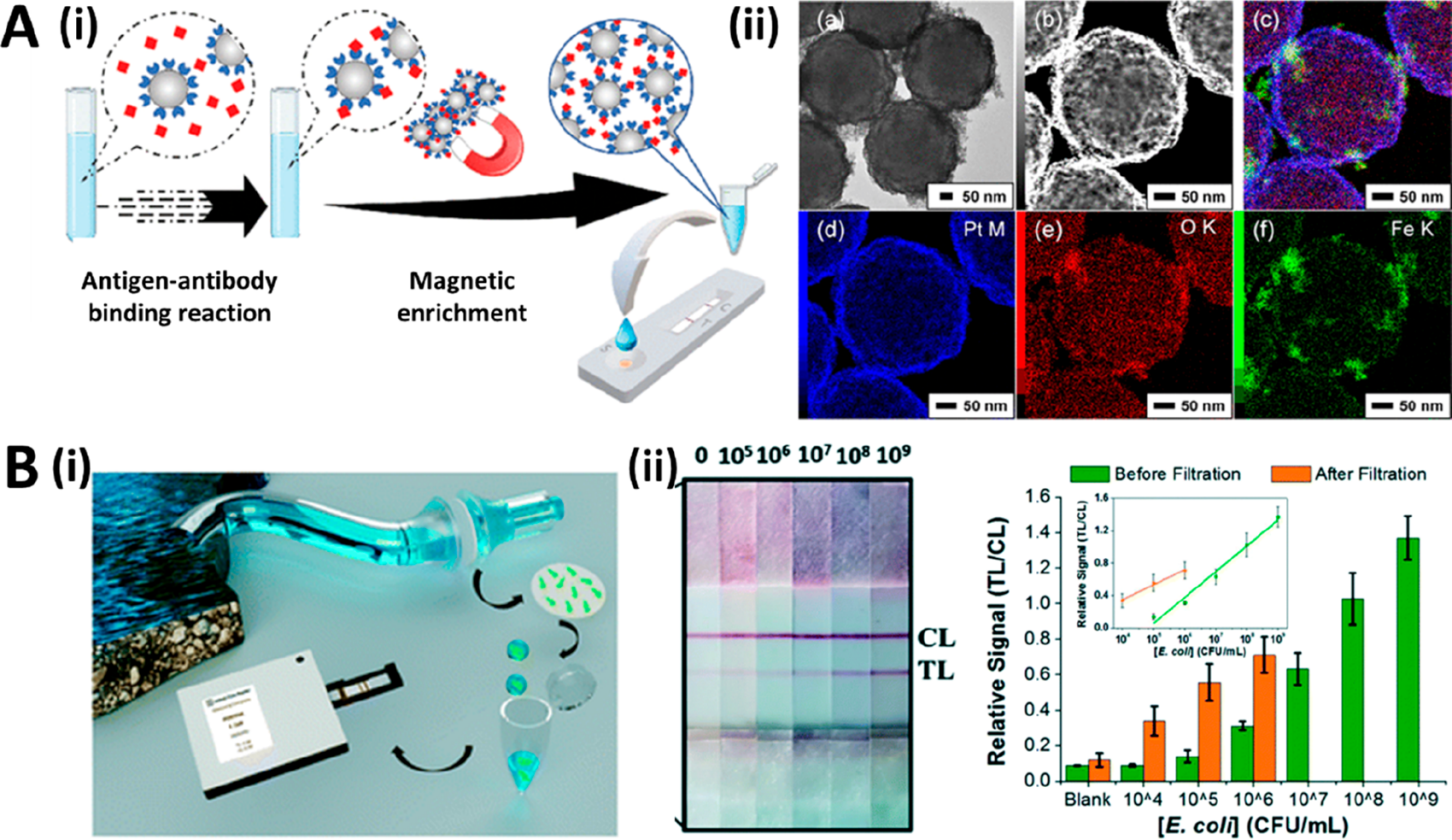
(A, i) Schematic representation of a sensitivity enhancement approach based on sample preconcentration by magnetic focusing. (A, ii, a) TEM image, (b) HAADF-STEM image, and (c–f) EDS elemental mapping images of Pt–P2VP@SPION. Reprinted (adapted) with permission from ref (74). Copyright 2021 American Chemical Society. (B, i) Schematic representation of a sensitivity enhancement approach based on sample filtration. (B, ii) AuNP-based LFA approach for the detection of *E. coli* in tap water (0–10^9^ CFU mL^–1^). Reprinted (adapted) with permission from ref (10). Copyright 2021 The Royal Society of Chemistry.

The sensitivity of LFAs can be vastly improved by the preconcentration/purification of the target analyte present in the sample, which is usually present at low levels and in a sample matrix with other biomolecules capable of interfering with the assay. Sample enrichment can be performed using magnetic separation or filtration. The former has been applied by Ren et al.,^73^ who have used antibody-functionalized magnetic particles in order to concentrate valosin containing protein up to 10-fold, yielding a 4000-times greater sensitivity than the conventional LFA.^73^ Similarly, Son Le et al.^74^ have used superparamagnetic iron oxide nanoparticles (SPIONs) that provide a magnetic enrichment factor (φ) = 40, for the detection of C-reactive protein. This enables a 26-fold lower LoD (0.08 ng mL^–1^) compared to the conventional AuNP-based LFA (Figure 4A).^74^ Magnetic enrichment strategies have shown great performance in the lab over the past decade; however, their commercialization has been hindered by the fact that they usually require multiple washing steps that make the assay less user-friendly and more prone to error.

In the case of sample filtration, its use is limited to applications with large sample volumes, and only a representative amount of the sample is evaluated: for instance, the detection of *E. coli* in tap water. In this regard, Bergua et al. have developed a filtration system that uses a 0.25 μm pore size filter, a small peristaltic pump, and microfluidic tubes to filter 300 mL of water in 15 min. With this approach, the authors have improved the LoD by 1 order of magnitude to 10^4^ CFU mL^–1^ (Figure 4B).^10^ While this is an effective strategy, sample filtration relies on the use of external instrumentation and a time-consuming pretreatment of the sample. It is beneficial to integrate a sample preconcentration step into the LFA strip. Lee et al. have developed an LFA with a Nafion-based ion concentration polarization (ICP) preconcentrator integrated in the conjugate pad. ICP is an ion transport phenomenon that occurs when ions selectively pass through an ion exchange membrane. It enables a 15-fold sample preconcentration in just 8 min using a portable 9 V battery.^75^ With this approach they achieved a 26% sensitivity enhancement for the detection of β-hCG.

### 3.3 Increasing Bioreceptor Concentration

One of the most straightforward ways to improve the LFA’s sensitivity is to increase the concentration of the capture bioreceptor in the TL, as this will lead to a greater analyte binding efficiency. However, this approach is limited by the loading capacity of the nitrocellulose membranes, which is usually around 80–100 μg cm^–2^.^16^ Researchers are developing novel strategies that use nanomaterials with high surface-to-volume ratios to increase the immobilization density of bioreceptors. For instance, Pan et al. used immobilized AuNPs functionalized with antibodies to the test line. Simultaneously increasing the surface area and thereby antibody concentration at the test line, and the pre-existence of AuNP at the test line, meant the signal from nonimmobilized AuNPs from the conjugate pad was evident at lower target concentrations. With this method they were able to immobilize up to 2.2 mg mL^–1^ of antibodies at the TL and achieve a 100-fold sensitivity enhancement for the detection of *Cronobacter sakazakii* compared to the conventional LFA.^76^ In this kind of approach, the nanomaterial selection dictates the assay’s performance. The nanomaterial at the TL needs to have the following four properties: (1) It needs to be small enough to penetrate the pores of the nitrocellulose membrane. (2) The suspension should avoid coffee ring effects. (3) The nanomaterial should contain functional groups that enable the oriented conjugation of the antibodies. (4) The optical properties of the nanomaterial should not interfere with the detection signal.

### 3.4 Novel Signal Transducers

**Figure 5:**
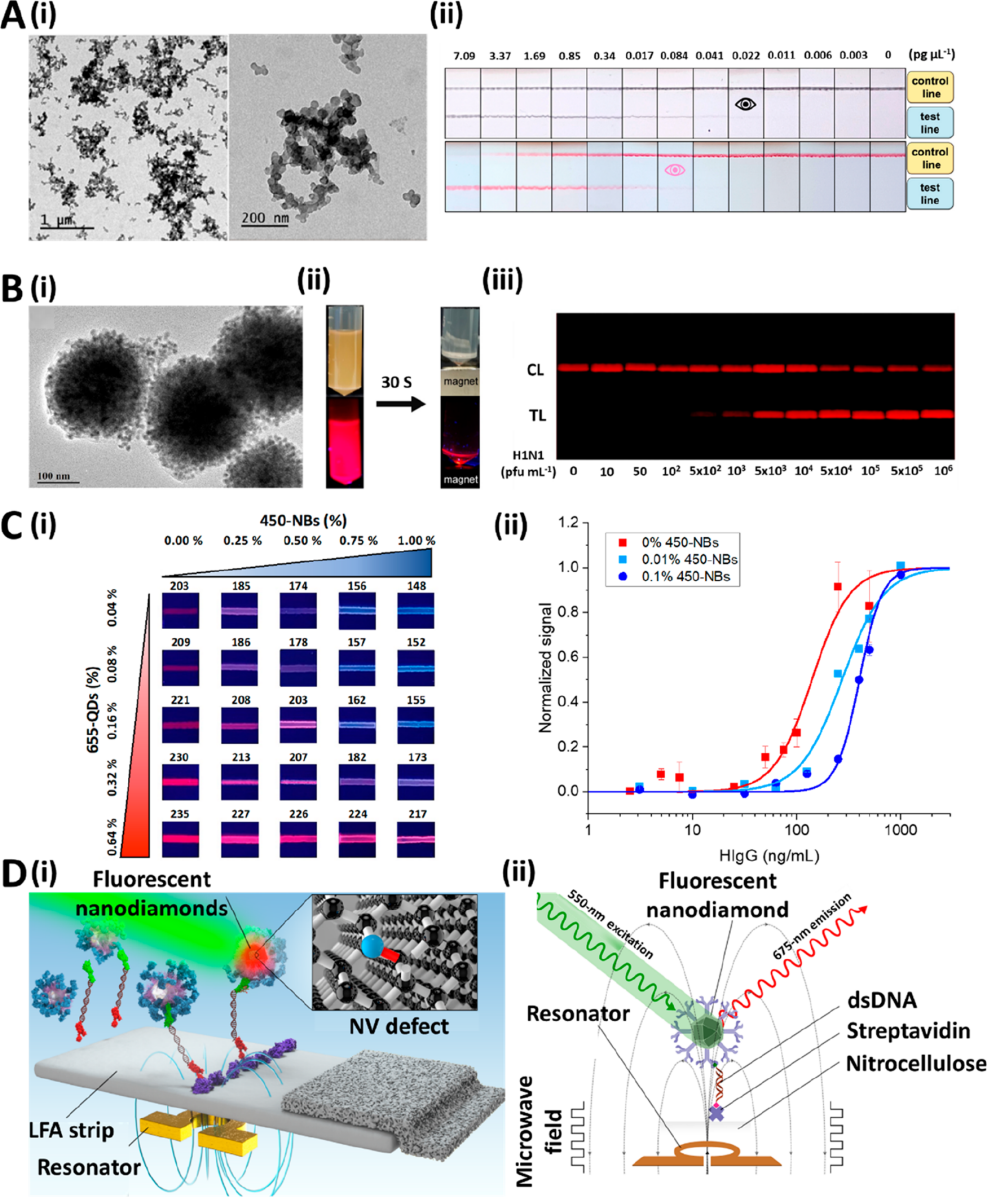
(A, i) TEM image of carbon nanoparticles that form aggregates of 10–100 nm. (A, ii) Photograph of LFA strips after detecting *E. coli* (0–7.09 pg μL^–1^) using carbon nanoparticles (black) and AuNPs (red). Reprinted (adapted) with permission from ref (77). Copyright 2021 MDPI. (B, i) TEM image of the magnetic QD nanobeads. (B, ii) Picture of the nanobeads before and after the magnetic enrichment step. (B, iii) Picture of the LFA strips after the detection of H1N1 (0–10^6^ pfu mL^–1^), showing an LoD of 500 pfu mL^–1^ by naked eye. Reprinted (adapted) with permission from ref (81). Copyright 2021 Elsevier. (C, i) Color palette generated in the TL of the LFA upon the ratiometric combination of different concentrations of 650-QDs (target-dependent) and 450-NBs (target-independent) reporters. (C, ii) Calibration curve for HIgG detection using LFA strips with increasing concentrations (0–0.1%) of 450-NBS in TL. Reprinted (adapted) with permission from ref (82). Copyright 2021 Wiley. (D, i) Schematic representation of a nanodiamond-based LFA for the detection of HIV-1 RNA. (D, ii) An omega-shaped stripline resonator is fixed under the LFA strip to selectively separate the fluorescence signal at the TL from the background autofluorescence. Reprinted (adapted) with permission from ref (84). Copyright 2020 Springer Nature.

Most commercial LFAs rely on the use of low-cost colorimetric AuNPs or dyed beads that quickly generate an optical signal that can be directly inspected by the naked eye. However, these are not suitable for scenarios where the target analyte is present at ultralow concentrations, mainly due to their low molar absorptivity, which means that a large accumulation of nanoparticles is required on the TL to generate a measurable optical signal. An alternative method of enhancing the sensitivity of LFAs is to use signal transducers with higher absorbances that create a stronger contrast with the background signal. Black carbon-based materials have garnered interest for this application. Porras et al. have reported a 3.8-fold lower LoD when using carbon nanoparticles compared to AuNPs, allowing the detection of *E. coli* in the nanomolar range by naked eye (Figure 5A).^77^ Similarly, Sun et al. have reported that the use of multiwalled carbon nanotubes (MWCNTs) in LFAs can provide a 10-fold sensitivity enhancement for methamphetamine detection compared to conventional AuNP-based LFAs.^78^ As well as providing an easily identifiable color change against the white background of the nitrocellulose, this nanomaterial possesses more binding sites than the AuNPs due to its greater surface area.

Over the past decade, fluorescence detection has become a common way of enhancing the sensitivity of LFAs. Quantum dots (QDs) are among the most used labels due to their narrow emission peaks and high quantum yield (high photon emission rate).^79^ In such systems it is possible to measure either the absorbance or the emission of the TL to calculate the analyte binding concentration; however, the emitted light is typically measured. The reason for this is that fluorescence measurements are more sensitive, mainly due to the way they are measured. While absorbance is measured over a bright background, fluorescence is measured without any reference beam and over a dark background.^80^ This enables the detection of low levels of light. Recently, Bai et al. have developed magnetic QD nanobeads by forming MnFe_2_O_4_ magnetic beads that they then coated with PEI. These positively charge nanoparticles with magnetic cores were coated with CdSe/ZnS to create 200 nm diameter superparamagnetic MnFe_2_O_4_ core nanoparticles with “quantum dot” coated surfaces. The multifunctional composite nanoparticles were shown to be capable of the enrichment and ultrasensitive detection of influenza A virions, capable of detecting as little as 22 pfu mL^–1^ in nasopharyngeal swabs (Figure 5B).^81^ Sena-Torralba et al. have developed a simple and cost-effective strategy based on the ratiometric combination of target-dependent red CdSe@ZnS QDs (650-QDs) and target-independent blue polystyrene nanobeads (450-NBs) in LFA (Figure 5Ci,ii). The approach enables the precise modulation of the assay’s dynamic range and sensitivity upon the use of different concentrations of both reporters. The authors prove a 78-fold higher sensitivity than the AuNP-based LFA when detecting HIgG and an 18-fold faster assay time (compared to ELISA).^82^ As sensitive as fluorescence-based detection methods are, their performance can be hindered by the intrinsic background fluorescence of nitrocellulose membranes. The choice of the optimal substrate (nitrocellulose, glass, etc.) when developing an LFA for an analyte depends on many of the material, chemical, and optical properties of the substrate, which must be tuned for the optical mechanism being used. For example, a substrate should be chosen that does not autofluoresce at the same wavelength as the desired fluorophore emits. A recent work by Shah and Yager sets out a quantitative framework to aid the selection of the appropriate substrate for LFA development.^83^

Other alternative fluorophores have also shown promise recently, most notably the use of fluorescent nanodiamonds by Miller et al. The emission intensity of these highly fluorescent nanoparticles is very high with emissions in the visible spectrum at (675 and 550 nm). The researchers have incorporated an omega-shaped stripline resonator under the LFA strip, which is used to apply a microwave-frequency electromagnetic field that selectively modulates the fluorescence signal of the nanodiamonds at a set frequency (Figure 5Di,ii). With this approach, the authors achieve sub-attomolar (8 × 10^–19^ M) limits of detection in a model biotin LFA, 10^5^ times more sensitive than the detection obtained with AuNPs. Additionally, they demonstrate the clinical utility of this strategy by proving the detection of HIV-1 RNA in real plasma samples after 10 min of isothermal amplification.^84^ Despite the recent advances in fluorescent LFA sensors, few have been commercialized, perhaps because they require instrumentation that conventional LFAs do not (excitation source, dark illumination, photodetector, etc.). To address this, portable versions of this equipment is being developed: such as phone-based fluorometers^85^ or affordable infrared laser diodes for the excitation of upconverting nanoparticles (UCNPs) (section 2).^86^

### 3.5 Signal Amplification

**Figure 6:**
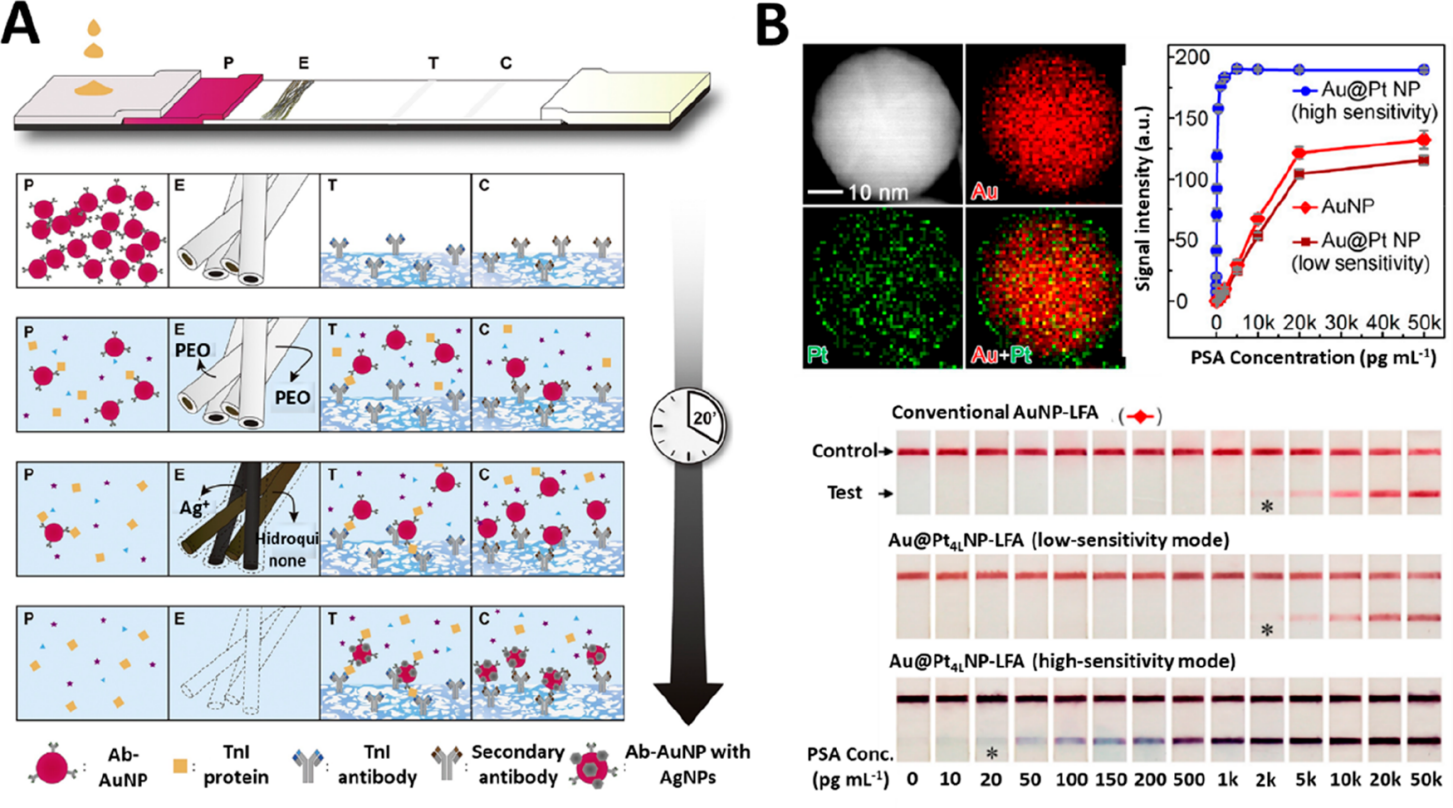
(A) Schematic representation of the signal amplification strategy using water-soluble nanofibers and the silver enhancement reaction. Reprinted (adapted) with permission from ref (88). Copyright 2018 Elsevier. (B) Signal amplification strategy using AuNPs coated with Pt ultrathin layers (Au@Pt_4L_), for the detection of PSA. Reprinted (adapted) with permission from ref (91). Copyright 2017 American Chemical Society.

Signal amplification is also a well-known strategy for sensitivity enhancement in LFA. There are several ways to amplify the signal at the test line, the most widely reported of which are AuNP enlargement through silver staining, or coating the AuNPs with enzymes or catalytic metals.^87^ Silver staining has been applied to the detection of Troponin I by the integration of water-soluble hybrid nanofibers between the conjugate pad and the TL. Once the AuNPs are bound at the TL, the silver enhancement reagents are released from the nanofibers and are reduced to metallic silver around the AuNPs (Figure 6A). The darkening of the TL enables a 10-fold sensitivity enhancement.^88^ The integration of the signal amplification reagents in the LFA strip is the most interesting advancement of this approach, since the speed and simplicity of use of the LFA is not compromised, but the signal is enhanced.

Enzymatic signal enhancement typically uses horseradish peroxidase (HRP) or alkaline phosphatase (ALP). These enzymes are traditionally used in ELISA and are commercially available, stable, and cheap. Parolo et al. achieved a 1 order of magnitude sensitivity enhancement in the detection of HIgG by the conjugation of AuNPs with anti-HIgG and HRP. The HRP catalyzed oxidation of 3,3′,5,5′-tetramethylbenzidine (TMB) was found to induce the best color change when compared to other methods, as would be expected.^89^ Unfortunately this assay required multiple washing steps, which hinders its applicability at the PoC. Moreover, it is reported that the presence of HRP inhibitors or competitors in the real sample matrices impedes these LFAs. Panferov et al. functionalized AuNPs with ALP and obtained a 10-fold sensitivity enhancement for the detection of potato virus X in leaf extracts. The authors claim that the ALP catalyzed LFA retains all the advantages of conventional LFAs: 15 min assay time, no additional equipment, no extra washing steps, and all the components able to be stored in the dry state.^90^

The signal amplification can also be performed by catalytic metals such as Pt, thin layers of which can be coated on AuNPs. Pt has a peroxidase-like catalytic activity toward the oxidation of TMB, so it can generate a blue TL. This approach enables a 100 times sensitivity enhancement for the detection of PSA, due to the large molar extinction coefficient of the oxidized TMB (Figure 6B). The authors also investigated the feasibility of the strategy for human plasma sample analysis, demonstrating that the complex sample matrix did not impair the catalytic activity of the Pt shells.^91^ Similarly, Loynachan et al. have taken advantage of the high catalytic activity of porous platinum core–shell nanocatalysts (PtNCs) to achieve an ultralow limit of detection of 0.8 pg mL^–1^ for p24 protein by naked eye.^92^

### 3.6 Alternative Signal Transduction Methods

In recent years, researchers have made efforts to develop alternative signal transduction methods in LFAs, to address the fact that current optical readers and sensing methods are insufficiently sensitive and precise enough for some clinical applications. While these alternative signal transduction methods [thermal contrast amplification (TCA), photoacoustic (PA) analysis, and surface-enhanced Raman scattering (SERS)] have been successfully utilized in other biosensing platforms,^93−95^ the goal has been to integrate these sophisticated detection systems into the simple paper-based architecture of LFAs. The development of PoC readers using user-friendly software to simply and quickly obtain and interpret assay results is discussed in section 2; in this section, we will simply discuss advances in the methodology of different signal transduction methods.

Thermal contrast amplification (TCA) is based on the measurement of the temperature change with an infrared camera, when irradiating the metal nanoparticles present on the test line with a near-infrared laser. The metal nanoparticles are used as light-to-heat transducers by adjusting the laser wavelength within the localized surface plasmon resonance (LSPR) peak of the nanoparticles. With this signal transduction principle and the relatively high resolution of infrared cameras (0.1 °C),^96^ the TCA approach enhances the sensitivity while simultaneously allowing precise quantification in LFAs. For instance, Wang and co-workers observed an 8-fold sensitivity enhancement for the detection of influenza, malaria, and *Clostridioides difficile*, compared to the optical signal provided by AuNPs.^57^ Similarly, Zhan et al. achieved an LoD of 8 pg/mL for the detection of p24 protein spiked into human serum,^97^ and Qu et al. obtained a 12-fold lower LoD for HCG biomarker compared with the visual detection mode.^58^ This detection method requires a careful calibration to compensate for background environmental temperature fluctuations.

Photoacoustic (PA) imaging is based on the measurement of acoustic waves generated after the irradiation of metallic nanoparticles with a laser. The PA signal transduction method includes three steps: absorption of the light by the AuNPs, the conversion of the absorbed energy into heat, and the heat-induced thermal expansion of the air that generates pressure oscillations.^22^ PA imaging provides outstanding sensitivity compared to optical imaging due to its ability to penetrate deeper into the nitrocellulose membrane. There is also a greater signal-to-noise ratio, due to the input (light) and output (acoustics) being different energy types. To date, there is only one reported use of PA in an LFA. This was reported by Zhao et al., who improved the sensitivity 100-fold when detecting cryptococcal antigen (CrAg), compared to the visible signal provided by the same AuNPs.^98^ As suggested by the authors, a barrier to the adoption of this technique might be the high price of the PA detector and oscilloscope. Hence, researchers are working on the development of portable and cost-effective alternatives such as replacing the laser with LEDs.

**Figure 7:**
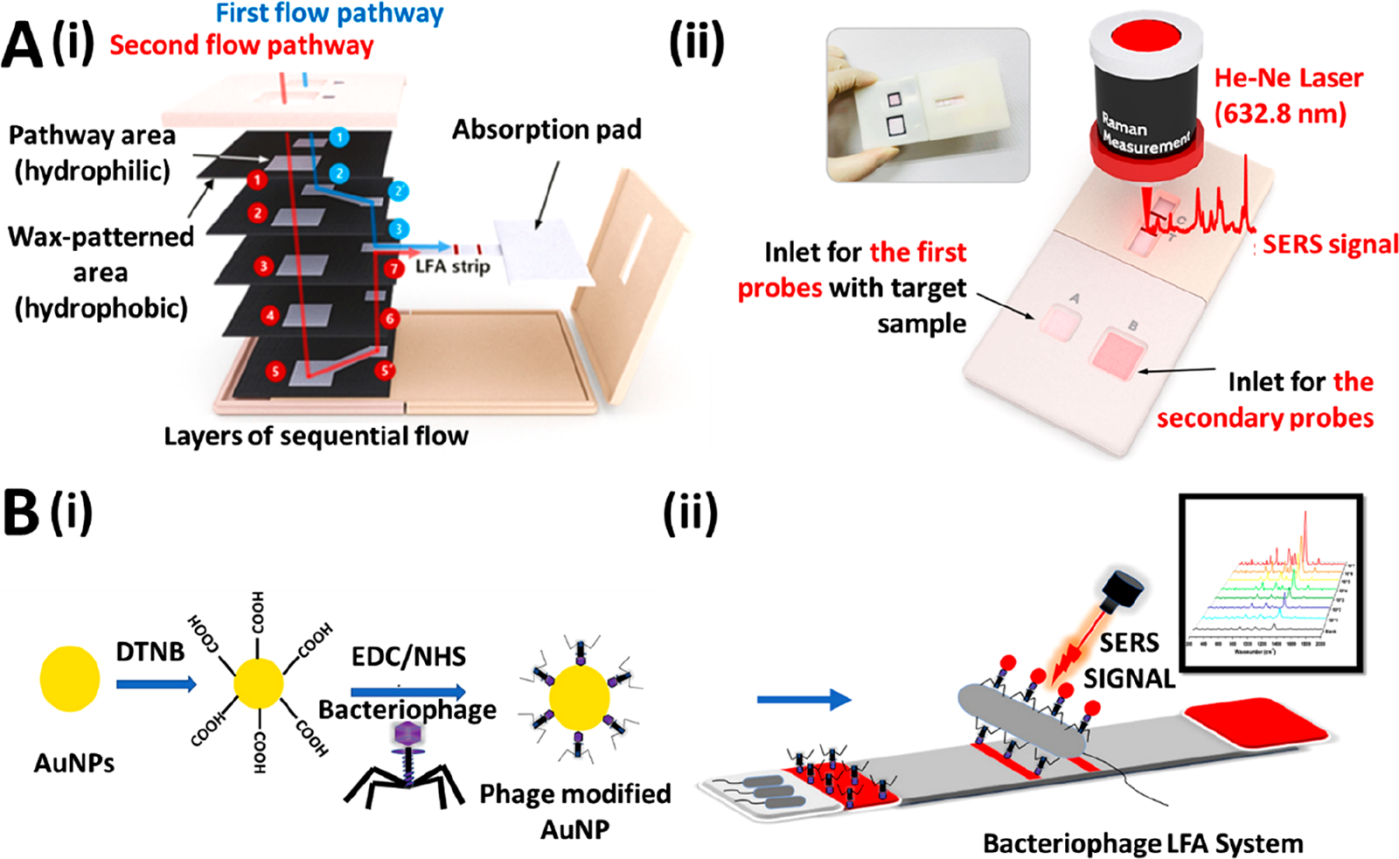
(A, i) Schematic representation of a dual-flow LFA fabricated using 5 wax patterned nitrocellulose membrane layers. (A, ii) 25 and 45 nm AuNPs are introduced into the first and second inlet, respectively. This sequential addition of differently sized AuNPs allows for reproducible SERS signal enhancement on the TL. Reprinted (adapted) with permission from ref (104). Copyright 2021 American Chemical Society. (B, i) A bacteriophage covalently coupled to AuNPs by carbodiimide coupling is used as a highly specific bioreceptor. (B, ii) The Bacteriophage LFA system enables the quantitative detection of *S. enteritidis* using SERS. Reprinted (adapted) with permission from ref (105). Copyright 2021 Elsevier.

The integration of surface-enhanced Raman scattering (SERS) into LFAs has been extensively reported during the past decade as a sensitivity enhancement strategy.^99−101^ It is based on the ability of metallic nanoparticles to enhance Raman signals.^102^ More recently, researchers have focused on further enhancing the analytical properties of LFAs by combining the SERS signal transduction with other strategies. For instance, Wang and colleagues have developed a SERS-based LFA using Fe_3_O_4_@Ag magnetic nanoparticles loaded with DTNB dyes for dual magnetic sample preconcentration and SERS signal generation. With this strategy, they have achieved a 2000-fold sensitivity enhancement for the detection of H1N1 and HAdV viruses, compared to the conventional AuNP-based LFA.^103^ Kim et al. have applied SERS on a dual-flow LFA, which was fabricated using 3-dimensionally stacked layers of wax-patterned nitrocellulose membranes (Figure 7A). The smaller 25 nm AuNPs used in this test were functionalized with anti-TSH antibodies and backfilled with biotinylated BSA (TSH, thyroid stimulating hormone). The biotin was then able to bind to streptavidin coatings on 45 nm AuNP that were flown over the TL in a secondary flow step. This caused an increase in SERS signal through electromagnetic enhancement effects. The authors note that the enhancement effect increases as the AuNP size increases, and simulations showed that using 45 nm AuNPs in both flow channels would give a greater SERS enhancement. However, the 25 nm AuNPs are more homogeneously distributed on the TL, which reduces the variation between test strips. With this approach, the authors achieve an LoD of 0.15 μIU/mL for the detection of TSH, with 0.5 μIU/mL being the threshold value for the diagnosis of hyperthyroidism.^104^ Finally, Ilhan et al. have used SERS signal transduction in combination with bacteriophages as bioreceptors. Bacteriophages are viruses that specifically infect bacterial species (Figure 7B). The authors propose their use in LFA as an alternative to antibodies, which are time-consuming and costly to produce. The bacteriophage LFA system enabled an LoD of 7 CFU/mL and exhibited similar analytical performance to an antibody-based LFA for the detection of *Salmonella enteritidis*.^105^

## 4 Multiplexing

Given the complex physiology of many pathological states (e.g., cancer), which produce multiple different biomarkers, the ratios and concentrations of which can be indicative of disease progression,^106^ diagnostic tests should ideally measure as many clinically relevant biomarkers as possible. Information about different biomarkers can inform clinical decisions based on the needs of individual patients.^107^ This need is also extended to food, environmental, and safety applications, where the assay’s utility increases with the number of targets detected (e.g., detecting multiple pollutants at once would speed up the analysis of water quality). Multiplexing is the simultaneous analysis of several analytes in a single sample and has been investigated for decades given its obvious time and sample saving potential.^108−110^ Considering that most diagnostic samples are extracted in limited volumes (e.g., blood and nasopharyngeal swab), multiplexing saves sample volume, time, and cost.^111,112^

Despite the many potential advantages that multiplexing offers, adding any complexity to the system inevitably leads to practical difficulties that must be overcome. When developing multiplexed LFAs there a several biological and physical issues that need to be resolved.^20^ One major challenge is the simultaneous detection of analytes present in the sample at very different concentrations (e.g., fM and μM),^113^ as sensors are developed and designed to work across a clinically relevant range. This has led to the development of cost-effective and simple strategies that allow the modulation of the assay’s dynamic range. Likewise, it is not always easy to resolve the signal from the detection of each of the biomarkers under investigation, which is usually solved by using different detection methods (e.g., fluorophores) for each biomarker; however, the optimization of the rest of the LFA may not be ideal for both biomarkers. Data handling has also appeared as a bottleneck when using multiplexed sensors. This has resulted in an increase in papers in recent years focusing on the development of software for the analysis and interpretation of large data sets in multiplexed diagnostics.^114^

In this section, we will review recent advances and technologies that have been developed to address the aforementioned challenges in multiplexed LFA development. These involve the design of new LFA architectures/functionalities or the use of novel signal transduction mechanisms (Table 3). Attention will be drawn to those methods that enable PoC and high-throughput multiplexing with ultrahigh sensitivity as well as those that are feasible for commercialization.

**Table 3:** Comparison of the Multiplexing Strategies Reported for LFA

strategy	signal transducer	target analytes	detection of	sample	sensitivity enhancement	ref
multiple TLs	SiO_2_@DQD	2	SARS-Cov-2-related HIgG and HIgM	human serum	10^4^-fold	(117)
multiple TLs	AuNPs	3	miRNA-21, miRNA-155, and miRNA-210	human serum	no	(118)
multiple TLs	AuNPs	3	*Giardia*, *Cryptosporidium*, and *Entamoeba*	human stool	no	(119)
multiple TLs	AuNPs	3	HIV, HCV, and HAV antibodies	human serum	no	(121)
multiple TLs and 2 labels	FAM and ROX fluorophores	13	HPV (16, 18, 31, 33, 35, 39, 45, 51, 52, 56, 58, 59, and 68)	human cervical swab	no	(122)
multiple dots	AuNPs	7	DNA alleles (FY*01, FY*02, FY02N.01, GYPB*03, GYPB*04, JK*01, and JK*02) related to four blood group SNPs	human whole blood	no	(125)
microarray	AuNPs	4	morphine, amphetamine, methamphetamine, and benzoylecgonine	human urine	no	(126)
multiple strips	UCP nanoparticles	10	*E. coli* O157:H7, *S. paratyphi A*, *S. paratyphi B*, *S. paratyphi C*, *S. typhi*, *S. enteritidis*, *S. choleraesuis*, *V. cholera O1*, *V. cholera O139*, and *V. parahemolyticus*	food samples	no	(128)
wax-patterned multichannel	catalytic signal amplification of AuNPs	3	*Clostridioides difficile* toxins A and B, and glutamate dehydrogenase (GDH)	human stool	8-fold	(129)
multiple strips	magnetic nanolabels	3	BoNT-A, BoNT-B, and BoNT-E	milk, apple, and orange juices	1000-fold	(56)
single TL and 3 labels	orange, red, and green AgNPs	3	DENV NS1 protein, YFV NS1 protein, and ZEBOV glycoprotein		no	(135)
single TL and 3 labels	Ag^NBA^@Au, Ag^MB^@Au, and Ag^R6G^@Au SERS nanotags	3	CK-MB, cTnI, and Myo cardiac biomarkers	human serum	100-fold	(136)
multiple TLs and 2 labels	red and blue latex beads	4	IgG and IgM specific to DENV and CHIKV	human whole blood	no	(130)
multiple TLs and 3 labels	AgNPs, spherical and desert-rose-like AuNPs	3	casein, ovalbumin, and hazelnut allergenic proteins	biscuits	no	(131)
multiple TLs and 2 labels	ZrMOF@CdTe NPs	2	heart-type fatty acid binding protein (h-FABP) and cardiac troponin (cTnT)	human serum	10-fold	(133)
multiple TLs	lanthanide-doped nanoparticles (YVO_4_: Eu 40%)	3	staphylococcal enterotoxins SEG, SEH, and SEI	PBS buffer	100-fold	(134)
multiple TLs	Ag^NBA^@Au SERS NPs	3	Myoglobin, cTn1 & CK-MB	human serum	1000-fold	(123)
multiple TLs	AuNPs labelled with malachite green isothiocyanate (MGITC)	2	*Clostridium difficile* surface layer protein A (SlpA) and toxin B (ToxB)	human stool	1000-fold	(124)

### 4.1 Multiplexing Enabled by Architecture Modifications of the LFA Strip

#### 4.1.1 Several Detection Lines in Parallel

The most obvious and simple way to perform multiplexing in LFA is by creating multiple different test lines (TLs) on the detection zone, with each TL responsible for the biorecognition of a different analyte. Most multiplexing approaches that adopt this methodology report the detection of up to three different target analytes. The limited length of the detection zone of the strip, usually around 4 cm, limits the number of TLs that can be incorporated into the LFA.^15^ The first TL must be fixed at a proper distance from the conjugate pad so as to provide enough time for the first biorecognition event to occur between the conjugated label and the target analyte.^70^ This distance should be optimized during the development of each LFA but is rarely less than 2 cm. Additionally, the distance between TLs should be at least 2 mm to allow the simple error-free readout by eye.^115^ Coupled with the inclusion of a control line (CL), there is an obvious limit on the number of TLs that can be incorporated into a standard LFA. An obvious solution would be to elongate the LFA strip; however, this causes an exponential increase in assay time; as described by the Washburn equation.^116^

**Figure 8:**
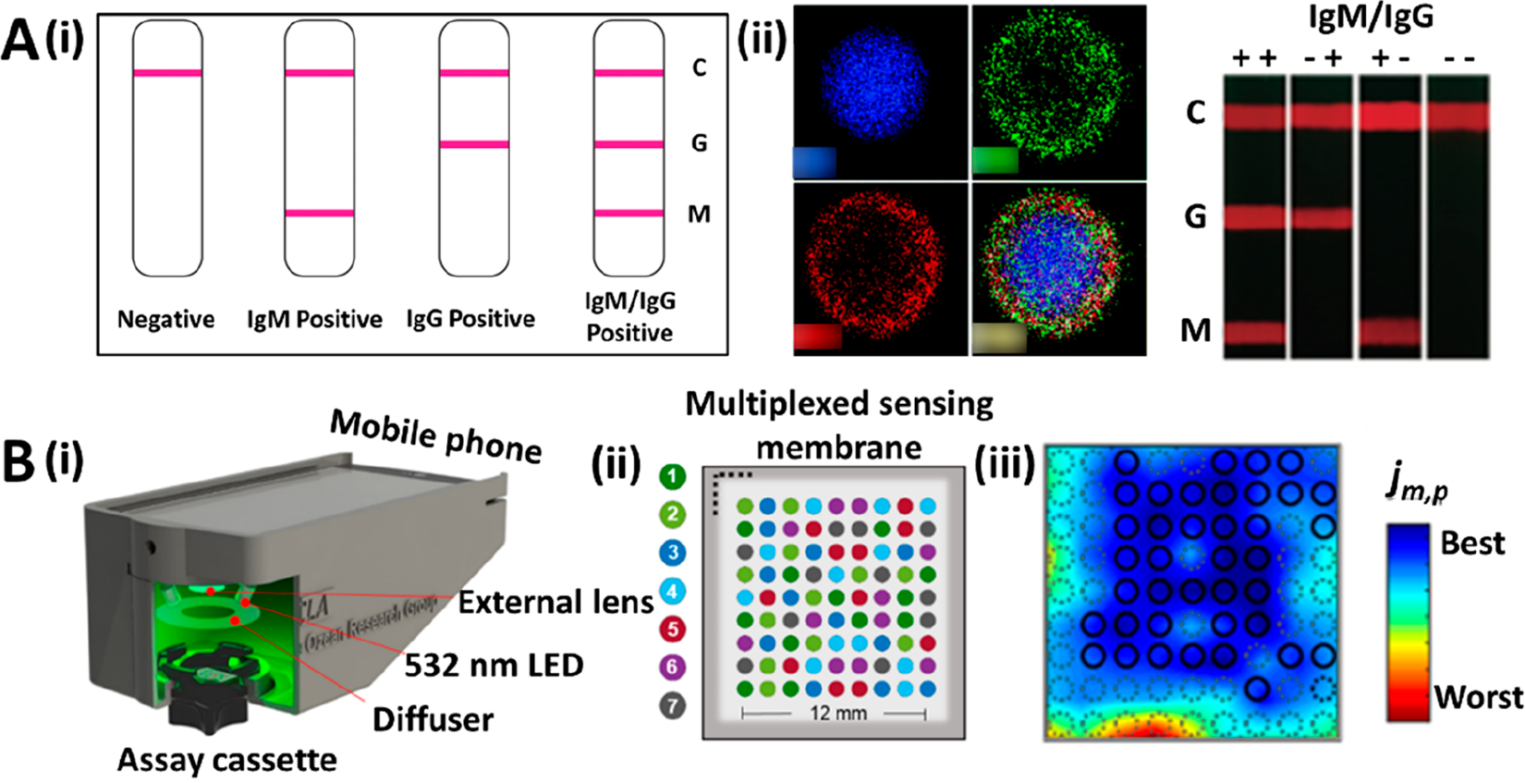
(A, i) Schematic representation of a multiplexed assay based on two consecutive TLs for the detection of IgM and IgG for SARS-Cov-2 spike proteins. (A, ii) Elemental mapping images of the SiO_2_@DQD used as fluorescent labels in the publication by Wang et al. (A, iii) Picture of the LFA strips after performing the assay for the detection of SARS-cov-2 antibodies, where the red fluorescence signal generated in the M, G, and C lines indicates the presence of IgM (M) or IgG (G), with a positive control (C). Reprinted (adapted) with permission from ref (117). Copyright 2021 The Royal Society of Chemistry. (B, i) Schematic representation of the mobile phone reader used to quantitatively evaluate multiplexed LFA microarrays. (B, ii) Picture of the algorithmically determined immunoreaction spot layout, where each row corresponds to a different spotting condition (1–7). (B, iii) Heat map of the microarray used to optimize the spot configuration by machine learning. Reprinted (adapted) with permission from ref (127). Copyright 2020 Springer Nature.

Wang et al. have developed a multiplexed and highly sensitive fluorescent LFA for the detection of SARS-CoV-2-related IgG and IgM. They fixed secondary antibodies specific to human IgM and IgG on the TL1 and TL2, respectively (Figure 8Ai); in the conjugate pad, they had 200 nm diameter silica-core@dual quantum dot (QD)–shell nanotags (SiO_2_@DQD) (Ems. 615 nm, Exc. 365 nm) functionalized with SARS-CoV-2 spike proteins (Figure 8Aii). The efficacy of this multiplexed LFA was established by the simultaneous detection of IgG and IgM from SARS-cov-2 positive and negative patient serum samples.^117^ Zheng et al. developed an LFA with three TLs for the simultaneous detection of micro-RNA-21, micro-RNA-155, and micro-RNA-210 cancer biomarkers in human serum. In such tests, the specificity of the probe sequences is vital for proper assay function, to prevent false positive results from nonspecific hybridization/binding.^118^ Multiplexed nucleic acid biomarker detection in LFAs has also been reported by Crannell et al. for the detection of DNA sequences specific to *Giardia*, *Cryptosporidium*, and *Entamoeba* protozoa in stool samples, all of which are usually present at very low concentrations. To improve assay sensitivity, this work used an isothermal amplification method (recombinase polymerase amplification, RPA) to generate more of the DNA sequence of interest, achieving an ultralow LoD of 444, 6, and 9 parasites/test for *Giardia*, *Cryptosporidium*, and *Entamoeba*, respectively.^119^ Many isothermal amplification technologies exist and are being investigated for use in LFAs; however, to date, most publications do not perform the amplification in the test strips but rather *ex situ* prior to running the assay. Likewise, it is common for papers to exclude the amplification time from the assay time to result, which they often increase substantially.

Lee et al. have developed a triple-TL LFA with a 100% clinical sensitivity and specificity for the detection of antibodies specific to HIV, hepatitis A, and hepatitis C antigenic peptides in patient sera. They have achieved this by immobilizing “proteinticles” in the TL, which hold the bioreceptors (antigenic peptides) on the nitrocellulose surface in a homogeneous orientation and conformation. “Proteinticles” are nanoscale protein particles that self-assemble inside cells with constant 3D structure and surface topology.^120^ In short, they are made by inserting the genetic sequence of the viral antigen peptide of interest to the C- or N-terminus of known proteins; for instance, in the work cited here by Lee et al., peptides from three viruses (HIV, HCV, and HAV) are inserted into the human ferritin heavy chain to create proteinticles.^121^ These are then expressed in bacteria to produce large protein complexes with structured, orientated, 3-dimensional scaffolds containing the antigen peptides being used as a bioreceptor in the assay. Proteinticles serve as an alternative to the direct fixation of the peptides on the TL, which normally suffers from limitations such as uncontrolled orientation, clustering, inactivation, or instability of the peptides, which can reduce the accuracy of the assay.^121^

The simplest method of multiplexing without extending the LFA strip length is to print test lines with two different bioreceptors that conjugate to analytes bound to different reporters (i.e., fluorophores that emit at different wavelengths). This method can be further multiplexed by printing multiple test lines, as has been shown by Xu et al.^122^ who have developed a multiplexed LFA for the simultaneous detection of the nucleic acid sequences specific to 13 different human papillomavirus (HPV) clades (16, 18, 31, 33, 35, 39, 45, 51, 52, 56, 58, 59, and 68). The researchers deposited 7 TLs, with 2 different bioreceptors in each TL, and used two fluorophores (FAM and ROX) as reporters. In this work, the nucleic acids were preamplified by linear-after-the-exponential (LATE)-PCR. As in all assays that preamplify the DNA, the sensitivity is determined by the amplification method; as such, they report an LoD of 10–10^2^ copies plasmid DNA/μL. They also report a high specificity with no cross-reactivity among 31 common HPV types.^122^

Multiple TLs need not only be used for optical readouts. Zhang et al. have taken advantage of the ultrasensitive signal and a wide linear dynamic range of SERS, to develop a multiplexed LFA for the detection of myoglobin, cTnI, and CK-MB cardiac biomarkers in human serum. The authors used SERS nanotags (silver core/gold shell NPs) loaded with Nile blue A dye (Ag^NBA^@Au) and a triple TL assay design. The assay displayed a linear response over the clinically relevant concentration ranges of the three biomarkers spanning over 6 orders of magnitude. They were able to obtain LoDs of 3.2, 0.4, and 0.5 pg mL^–1^ for myoglobin, cTnI, and CK-MB, respectively.^123^ Similarly, Hassanain et al. have utilized SERS for both the multiplexed and highly sensitive detection of *Clostridium difficile* surface layer protein A (SlpA) and toxin B (ToxB). In this work, the authors report the use of a handheld Raman spectrometer that enables the PoC quantification of the biomarkers down 0.01 pg μL^–1^.^124^

#### 4.1.2 Microarrays

To make better use of the test area afforded by lateral flow strips, rather than printing test lines, it is possible to make microarrays using test spots. Once example of this is the work published by Gomez-Martinez et al., who designed a 4 × 2 microarray for the simultaneous detection of 7 DNA alleles (FY*01, FY*02, FY02N.01, GYPB*03, GYPB*04, JK*01, and JK*02) for rapid blood group genotyping. The authors spotted 8 capture oligonucleotide probes on the test area of a single nitrocellulose membrane, with one of these being the positive control. While this approach successfully enabled a fast blood group genotyping (total processing time of 1 h), it did require a LATE-PCR preamplification step which increased the assay complexity and price, since extra instrumentation and reagents were required.^125^

In another work, Taranova et al. managed to deposit up to 32 spots in a microarray format (4 × 8) on the NC membrane. The spots had a diameter of 250 μm and were precisely deposited by dispensing 20 nL of the capture reagents with a steel pin and program-controlled manipulator. Rather than using this approach to detect 32 different analytes, they used the 8 spots in each row as replicates for the detection of 4 drugs (morphine, amphetamine, methamphetamine, and benzoylecgonine) in urine. Making repeat measurements in a single strip increases the robustness of the assay results, allowing for more accurate quantification. The authors claim that with the resolution of their setup, they have the capability to print arrays with up to 150 spots in the detection zone.^126^ This would be a large step forward in the development of high-throughput multiplexed LFAs. Ballard et al. have developed a machine-learning-based framework to determine the optimal configuration of the spots in the microarray and to quantify the analyte concentration. A mobile phone-based readout system that uses custom-built image processing software to analyze the microarrays has also been developed (Figure 8Bi). The algorithm is designed for assay optimization, designed to accurately select the best sensing conditions out of up to seven possibilities being tested in the microarray (Figure 8Bii). Additionally, the deep-learning framework developed enables the selection of the best spatial configuration of the immobilized spots (Figure 8Biii).^127^

#### 4.1.3 Combination of LFA Strips and Design of Innovative Paper Configurations

A quick and simple way to develop a multiplexed LFA is by connecting several test strips to a single sample pad. This has been reported by Zhao et al., who have developed a multiplex strategy based on a 10-channel lateral flow assay for the detection of 10 foodborne pathogens (*E. coli* O157:H7, *Salmonella paratyphi* A, *S. paratyphi* B, *S. paratyphi* C, *Salmonella typhi*, *S. enteritidis*, *Salmonella choleraesuis*, *Vibrio cholera* O1, *V. cholera* O139, and *Vibrio parahaemolyticus*). Each strip was functionalized with monoclonal antibodies specific to one of the pathogenic bacteria, and the conjugate pad was prepared by conjugating the antibodies with phosphor nanoparticles. The results obtained were 100% consistent when validated against the culture-based detection of samples taken from 279 food samples.^128^ Despite being fast and reliable, the use of this kind of multiplexing format is limited by the requirement of larger sample volumes. In this case, the sample volume required was 7 times higher (700 μL) than the single-strip LFAs. This prevents the use of this multiplexing method in scenarios where the sample volumes are prohibitively low.

**Figure 9:**
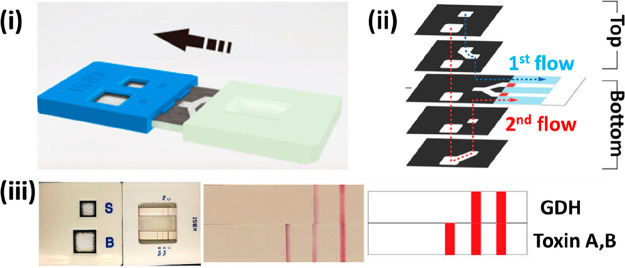
(i) Schematic representation of the multichannel device created by Kyoung Han et al. in which wax patterned-NC membranes were stacked in multiple layers (ii) to create flow paths that allow for the sequential flow of the sample and signal amplification reagents to the test line. (iii) Picture of the device after the detection of GDH and *Clostridioides difficile* toxins A and B. Reprinted (adapted) with permission from ref (129). Copyright 2021 Elsevier.

Han et al. have developed a similar strategy but, in this work, stack nitrocellulose sheets on top of each other, using wax patterning to create different flow paths vertically through the sheets (Figure 9i). This methodology was implemented to allow the sequential flow of signal amplification reagents to increase the flow sensitivity (Figure 9ii). With this approach, the authors achieved the simultaneous detection of *Clostridioides difficile* toxins A and B, and glutamate dehydrogenase (GDH) (Figure 9iii). The signal amplification strategy, based on the aggregation of AuNPs, enabled the detection of the target analytes at ultralow concentrations, with LoDs of 0.16, 0.09, and 0.03 ng mL^–1^ for GDH, *C. difficile* toxin A, and *C. difficile* toxin B, respectively.^129^

A multiplexing strategy based on the interrogation of three individual LFA strips using a single processor unit has been reported by Orlov et al. In this work magnetic nanoparticles were conjugated to monoclonal antibodies and used as labels, with each strip responsible for the detection of a particular target analyte. The assays were developed to detect *Botulinum* neurotoxins (BoNTs) A, B, and E in whole milk and juice samples and were simultaneously analyzed using a magnetic particle quantification (MPQ) reader.^56^ The use of this type of signal transduction enabled high assay sensitivity compared to the conventional colorimetric readout, allowing the detection of the target analytes in the low pg mL^–1^ range. The authors observe a linear range across 7 decades in this work which highlights the suitability of this method for multiplexed LFAs where analytes can span wide concentration ranges. Special attention should be given to the price, portability, and user-friendliness of the MPQ reader, as this might hinder the applicability of this platform in real scenarios.

### 4.2 Using Nanoparticles for Multiplexing

As has been seen throughout this Review so far, nanoparticles are routinely used in LFAs. By designing LFAs with different nanoparticles that generate different signals it is easy to envisage how multiplexed assays can be developed. In such systems it is important to be able to distinguish the signals quickly and easily from the different nanoparticles to allow the fast, efficient, and qualitative interpretation of the assay results. Recent research has also focused on the integration of low-cost, portable, and robust assay readout technologies. This section of the Review will focus on novel nanoparticle LFAs that have been developed for multiplexed sample analysis.

As well as AuNPs, latex beads are very popular nanoparticles for LFA development. An example of latex beads being used in a multiplexed LFA is the work of Lee et al., who developed a multiplex LFA that uses red and blue latex beads for the detection of IgG and IgM produced as a response to infections by dengue virus (DENV) and Chikungunya virus (CHIKV). The appearance of a purple color on the TL indicated the presence of both IgG and IgM antibodies in the sample. A quantitative evaluation of the strips was performed with a smartphone camera and image analysis software. In this work the hue (H) value was analyzed instead of the RGB value, as it was found to be more accurate.^130^ Similarly, Anfossi et al. used spherical AgNPs, spherical AuNPs, and “desert-rose-like” AuNPs, with SPR peaks at 420, 525, and 620 nm, respectively, to develop a multiplexed LFA for detection of casein, ovalbumin, and hazelnut allergenic proteins in commercial biscuits. The LFA comprises three lines, each responsive to one allergen. In order to obtain equivalent signal intensities from the different nanoparticles, the authors had to increase the concentration of AgNPs in the conjugate pad. This is not surprising since AgNPs have lower extinction coefficients than AuNPs. The results of this particular multiplexed assay are easy to interpret since the colors of each nanoparticle (cyan, yellow, and magenta) can be easily interpreted using conventional image analysis software.^131^

**Figure 10:**
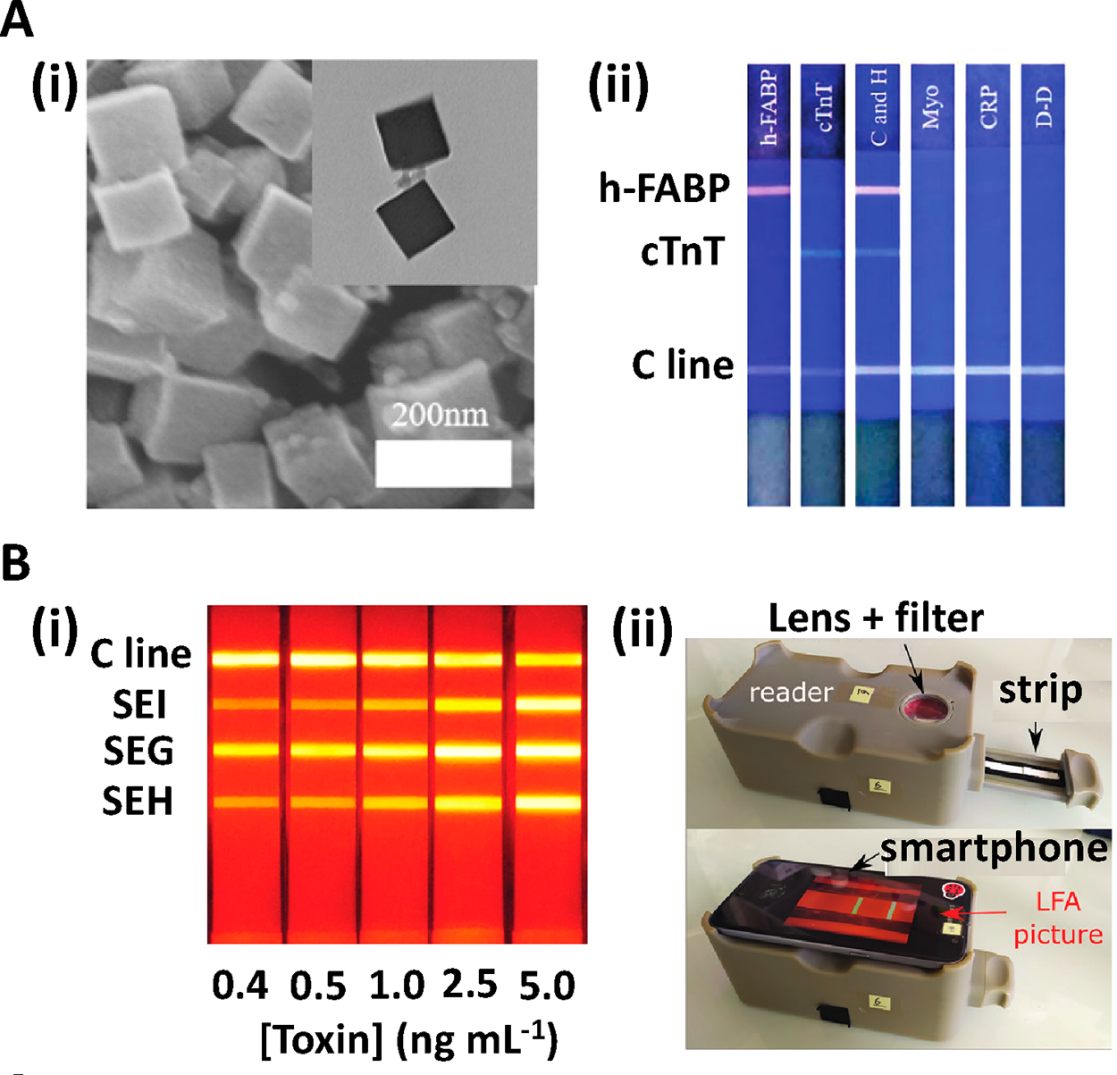
(A, i) SEM and TEM images of the ZrMOF@CdTe NPs, which show a cubic shape and a size around 120 nm. (A, ii) Multiplexed LFA based on the use of two TLs and ZrMOF@CdTe NPs with yellow and green fluorescent emissions. Reprinted (adapted) with permission from ref (133). Copyright 2021 The Royal Society of Chemistry. (B, i) Multiplexed LFAs showing the simultaneous detection of the staphylococcal enterotoxins I, G, and H, using lanthanide-doped nanoparticles. (B, ii) Smartphone-based readout system for the on-site LFA’s quantification. Reprinted (adapted) with permission from ref (134). Copyright 2021 The Royal Society of Chemistry.

The appropriateness of the colorimetric LFA is usually diminished by the low sensitivity and ease of quantification of this type of signal readout. Quantum dots (QDs) have advanced optical properties that make them ideal for the development of highly sensitive, easily quantifiable multiplexed LFAs. However, the fluorescence emission of QDs can be suddenly quenched by biomolecules present in biological samples, compromising the sensitivity and reliability of the assay.^132^ To resolve this, recent research has investigated the encapsulation of QDs into nanoparticles that shield them from quenchers. For instance, Zou et al. have reported the encapsulation of CdTe QDs into cubic zirconium metal organic frameworks (ZrMOFs; approximately 120 nm long) (Figure 10Ai), and their use as fluorescent labels for the simultaneous detection of heart-type fatty acid binding protein (h-FABP) and cardiac troponin T (cTnT). This nanomaterial is synthesized with a simple hydrothermal method, in which the reaction time can be modulated to obtain ZrMOF@CdTe NPs with different fluorescent properties. The authors have taken advantage of this to develop a multiplexed LFA that uses two TLs for ZrMOF@CdTe NPs that emit yellow and green light after being excited by a single wavelength (Figure 10Aii). This approach yielded visual LoDs of 1 and 250 μg L^–1^ for h-FABP and cTnT, respectively, below the lowest clinically relevant concentration. In addition, the researchers report a high specificity over other cardiac biomarkers [including D-dimer (D-D), myoglobin, and C-reactive protein (CRP)].^133^

Lanthanide-doped nanoparticles (YVO_4_/Eu 40%) have been shown by Mosseau et al. to possess remarkable optical properties compared to the QDs, due to an absence of photobleaching and longer luminescence times. One of the advantages of this work is the ease of nanoparticle synthesis which is achieved by the coprecipitation YVO_4_ and Eu salts. These nanoparticles were used for the simultaneous detection of staphylococcal enterotoxins G, H, and I, by the conjugation of the nanoparticles with monoclonal antibodies and the immobilization on three consecutive TLs in the detection pad (Figure 10Bi). This multiplexed assay was 100 times more sensitive than the comparative AuNP-based LFA, reaching LoDs in the pg mL^–1^ range for all the biomarkers investigated. Additionally, the researchers developed a portable and cost-effective smartphone-based readout system (see section 2; Figure 10Bii).^134^

#### 4.2.1 Multiplexing in a Single TL

**Figure 11:**
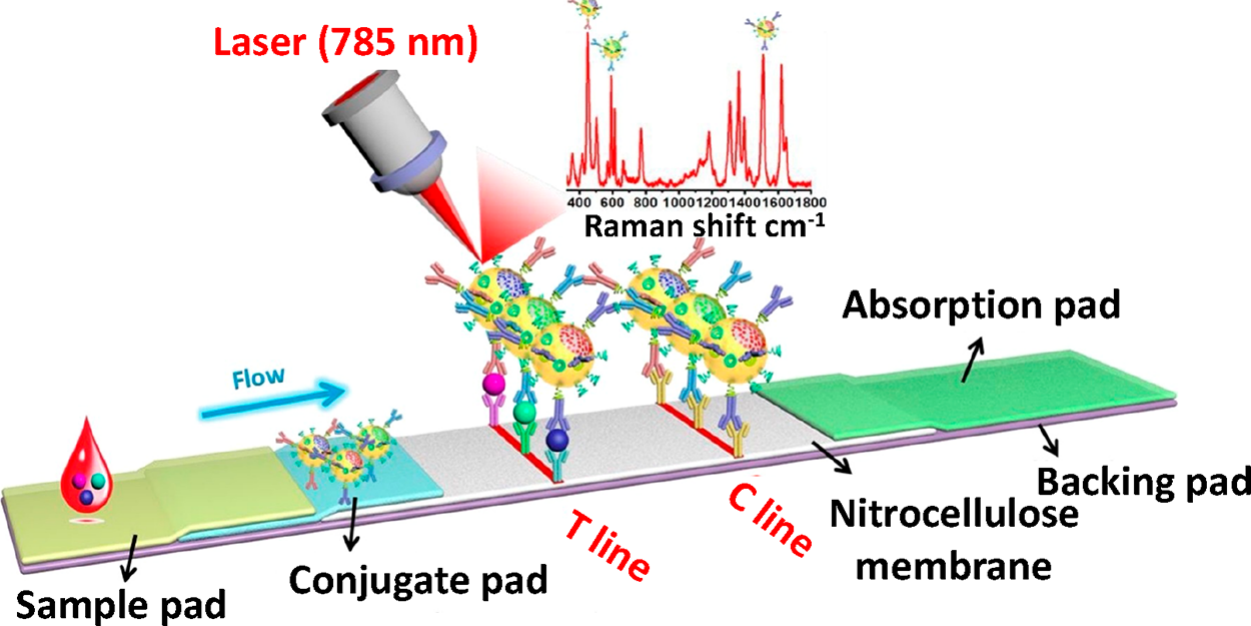
Multiplexing strategy based on the detection of CK-MB, cTnI, and myoglobin cardiac biomarkers in a single TL, using three Raman reporters. Reprinted (adapted) with permission from ref (136). Copyright 2018 Elsevier

The detection of different target analytes within a single TL is probably the most powerful method of multiplexing in LFAs. This retains the simplicity of a conventional double-line LFA, in terms of both fabrication and readout of results. Again, multiplexing in one TL can be coupled to printing several TLs to exponentially increase the multiplexing capability of an LFA. To perform multiplexed detection in a single TL, several labels with easily differentiated signals must be used. For instance, Yen et al. utilized the size-dependent optical properties of AgNPs to develop a multiplexed LFA for the detection of the dengue virus (DENV) NS1 protein, the yellow fever virus (YFV) NS1 protein, and an Ebola virus (ZEBOV) glycoprotein. The three monoclonal capture antibodies were deposited in equal concentrations on a single TL, and the conjugated pad was loaded with a mixture of orange, red, and green AgNPs, each functionalized for a separate target analyte. The presence of the three proteins in the sample turned the TL brown. The assay was shown to be quantifiable using RGB analysis with a smartphone camera.^135^ Similarly, Zhang et al. have developed a multiplexing strategy for the detection of three cardiac disease biomarkers for the early detection of acute myocardial infarctions [creatine kinase-MB isoenzymes (CK-MB), cardiac troponin I (cTnI), and myoglobin] in a single TL, using SERS signal transduction (Figure 11). In this work antibody-functionalized silver core and gold shell NPs loaded with three different Raman dyes [methylene blue (MB), Nile blue A (NBA), and rhodamine 6G (R6 G)] were used (Figure 11). The authors reported ultralow detection limits (in the low pg mL^–1^ range) and wide linear dynamic ranges (3 orders of magnitude) that cover the clinical ranges of the three biomarkers. This multiplexing approach is particularly useful for SERS, since Raman mapping can be time-consuming. In this work the time to result was reduced from 21 min for three consecutive TLs to 7 min for a single multiplexed test line.^136^

## 5 Evaluation of Complex Samples

One of the biggest challenges in LFA development is to achieve high analytical capabilities in complex real-world samples. This is a fundamental issue that must be resolved to upgrade the technology readiness level (TRL) to more than 4 (see section 6).^137^ Most real-world samples contain a multitude of components that can rapidly and drastically vary in composition; these variations alter the physicochemical properties of the assay.^138^ For example, the pH and ionic strength of the sample can affect the stability and binding constant of the bioreceptors to the target analyte.^139^ Therefore, any fluctuations of these in the sample can vastly affect the analyte capture and therefore assay quantification.

The optimal working conditions for most antibodies are at pH 7.4^15,140,141^ and ionic strengths of around 30 mM (although low ionic strength might also contribute to nonspecific protein–protein interactions).^142,143^ Nucleic-acid-based probes have an optimal working pH of 7.5–8.5^144^ and show better affinity at higher salt concentrations (>100 mM).^145−151^ It is important to know the pH and ionic strength of the media in which the LFA will be run as these can vary drastically, not just between media but also in a single media over time. For instance, saliva has a pH of 7.3–7.4 and ionic strength of 0.15 M,^152^ whereas sweat has a much lower pH of between 4 and 6 and an ionic strength that varies greatly with sweat rate, location on the body, and evaporation.^153,154^

One method to overcome this is to adjust the pH and ionic strength of the samples to the optimal assay conditions before performing the assay. This is done by drying a buffer into the sample pad of the LFA. There are physical as well as chemical properties of the sample that can affect the LFA; for example, the sample viscosity will affect the flow rate and in extreme conditions can even clog the nitrocellulose pores and prevent the sample from reaching the detection zone.^81^ Likewise the opacity of certain real samples such as whole blood^16^ can mask the optical signal generated at the TL. Common methods to overcome this include the incorporation of sample dilution or washing steps,^156^ often including anionic surfactants such as Tween-20^157^ and sodium dodecyl sulfate.^158^

The analytical sensitivity and specificity of LFA are frequently impaired when evaluating real samples due to the presence of analytes that are similar to the biomarker of interest. It is therefore vital to identify a bioreceptor that is highly specific to the biomarker of interest.^159^ Any assay is only going to be as good as the bioreceptor it uses. Assay performance can be improved by the incorporation of sample pretreatment methods such as analyte preconcentration (e.g., magnetic pre-enrichment^160^ and nucleic acid amplification^151,161^) or filtering.^162^ However, these usually increase the number of steps, reagents, ease-of-use, and time to result of the assay.

It is important to optimize all these conditions, especially when trying to improve the sensitivity of LFAs for the analysis of low-concentration biomarkers such as tau proteins (Alzheimer disease),^163^ soluble CD25 (hepatocellular carcinoma),^164^ and tumor necrosis factor-α (obstructive sleep apnea syndrome)^165^ that are present at trace levels (pg mL^–1^ range).

In this section of the Review, we will discuss recent advancements and novel methodologies to better the analytical performance of LFAs in complex media.

### 5.1 Sample Filtration

One of the most straightforward ways to purify the sample and preconcentrate the target analyte is by using external or integrated filtration membranes. At the time of writing, 0.45 μm pore diameter cellulose nitrate filters are routinely used for the preconcentration of bacteria in water samples.^166^ Integrated filters are also commonly used in LFAs that analyze whole blood samples, primarily for the removal of red blood cells. Two commercially available plasma separation membranes are Cytosep and Vivid plasma;^162,167^ examples for their use in LFAs include the detection of the capsid protein VP72 from the African swine fever virus^168^ and the diagnosis of SARS-CoV-2^169^ and antihuman immunodeficiency virus (HIV).^170^

**Figure 12:**
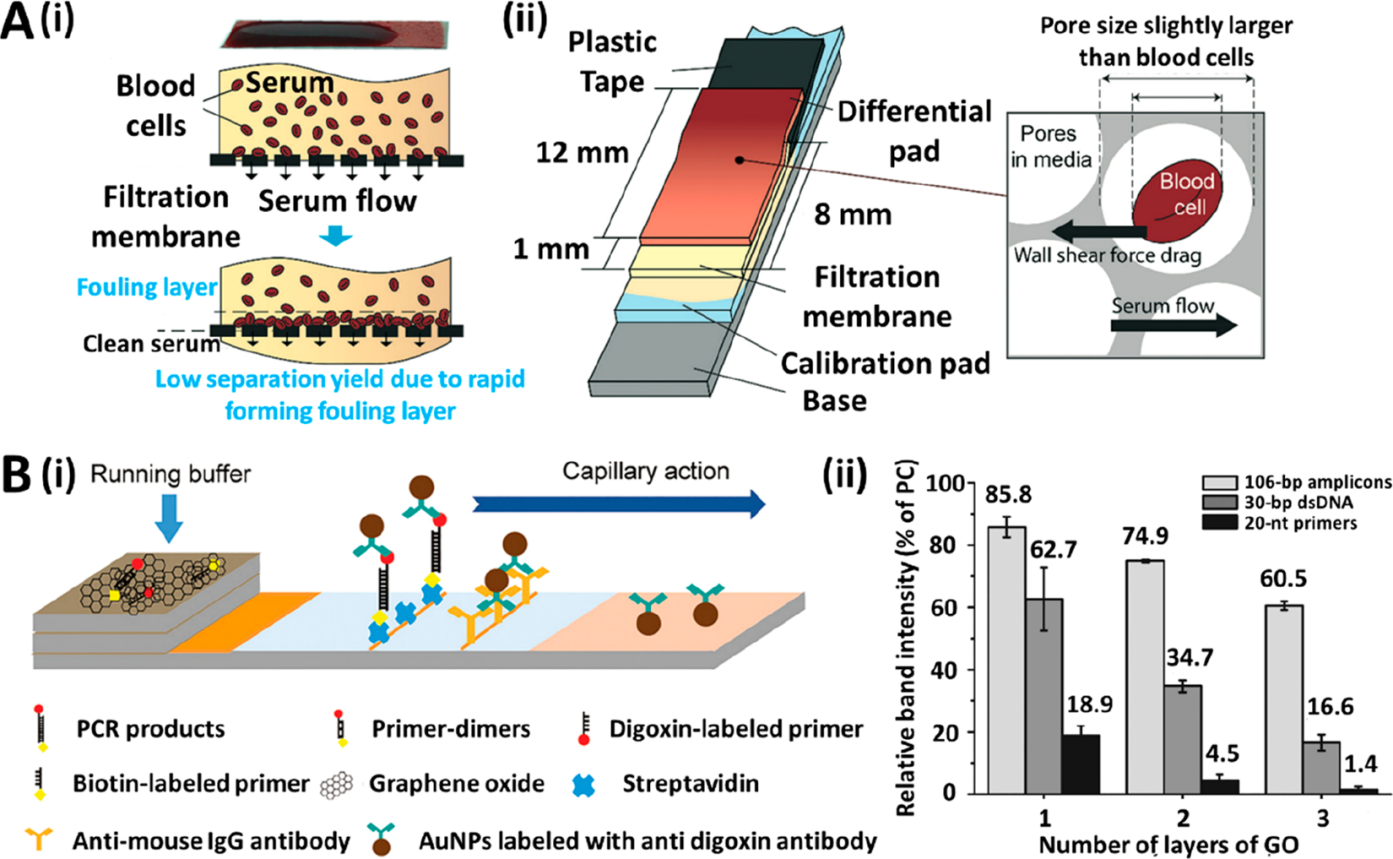
(A, i) Schematic representation of red blood cells clogging the filtration membrane. (A, ii) The use of the differential pad avoids the formation of the blood fouling layer, while the filtration and calibration pads are responsible for the separation and storage of the serum sample, respectively. Reprinted (adapted) with permission from ref (171). Copyright 2018 The Royal Society of Chemistry. (B, i) Schematic representation of the integrated PCR amplicon purification approach based on the immobilization of functionalized graphene oxide on the sample pad. (B, ii) The three-layered GO sample pad enables the scrubbing of almost all the residual primers and primer-dimers with <40 pb out of the sample matrix. Reprinted (adapted) with permission from ref (172). Copyright 2017 American Chemical Society.

One of the drawbacks to these types of filters is that the small pore sizes of the membranes (<9 μm) can promote blood clotting (Figure 12Ai), which limits the plasma/serum separation yield to just 10–30%.^171^ To this end, Lu et al. have developed a low-cost (<$0.10) cross-flow filtration pad that increases the separation yield by up to 86%. This method requires three paper-based membranes stacked on top of each other. The top “differentiation pad” (40 μm pore size) is responsible for preventing the formation of the blood fouling layer. The middle “filtration pad” (1.9 μm pore size) is responsible for filtering out the red blood cells, and the final “calibration” membrane (also 1.9 μm pore size) is used to store the sample (liquid holding capacity of 40 μL cm^–2^; Figure 12Aii). The authors have applied this approach for the multiplexed detection of CRP, RBP, and ferritin, achieving similar analytical accuracy as standard centrifuge-based serum separation methods.^171^

In certain situations, it is known what molecules will be present that need to be removed in order to improve the assay performance. In such situations, the filters can be functionalized to specifically remove known contaminants. One such example of sample scrubbing has been shown by Li et al., who have reported an innovative strategy to purify PCR amplicons directly in the LFA strip. They integrate high-surface-area graphene oxide (GO) functionalized for the specific capture of their PCR primers into the sample pad of their LFA (Figure 12Bi). They report the adsorption of almost all the residual primers, and primer-dimers with less than 40 bp, within the sample pad, while their 106 bp amplicons were able to flow to the test line (Figure 12Bii). With this approach, the authors achieved a 1000-fold sensitivity enhancement for the detection of bacteriophage λ-DNA, obtaining an LoD of 30 copies by naked eye.^172^

### 5.2 Sample Pretreatments Based on Active yet Automatic Manipulation of the Sample

**Figure 13:**
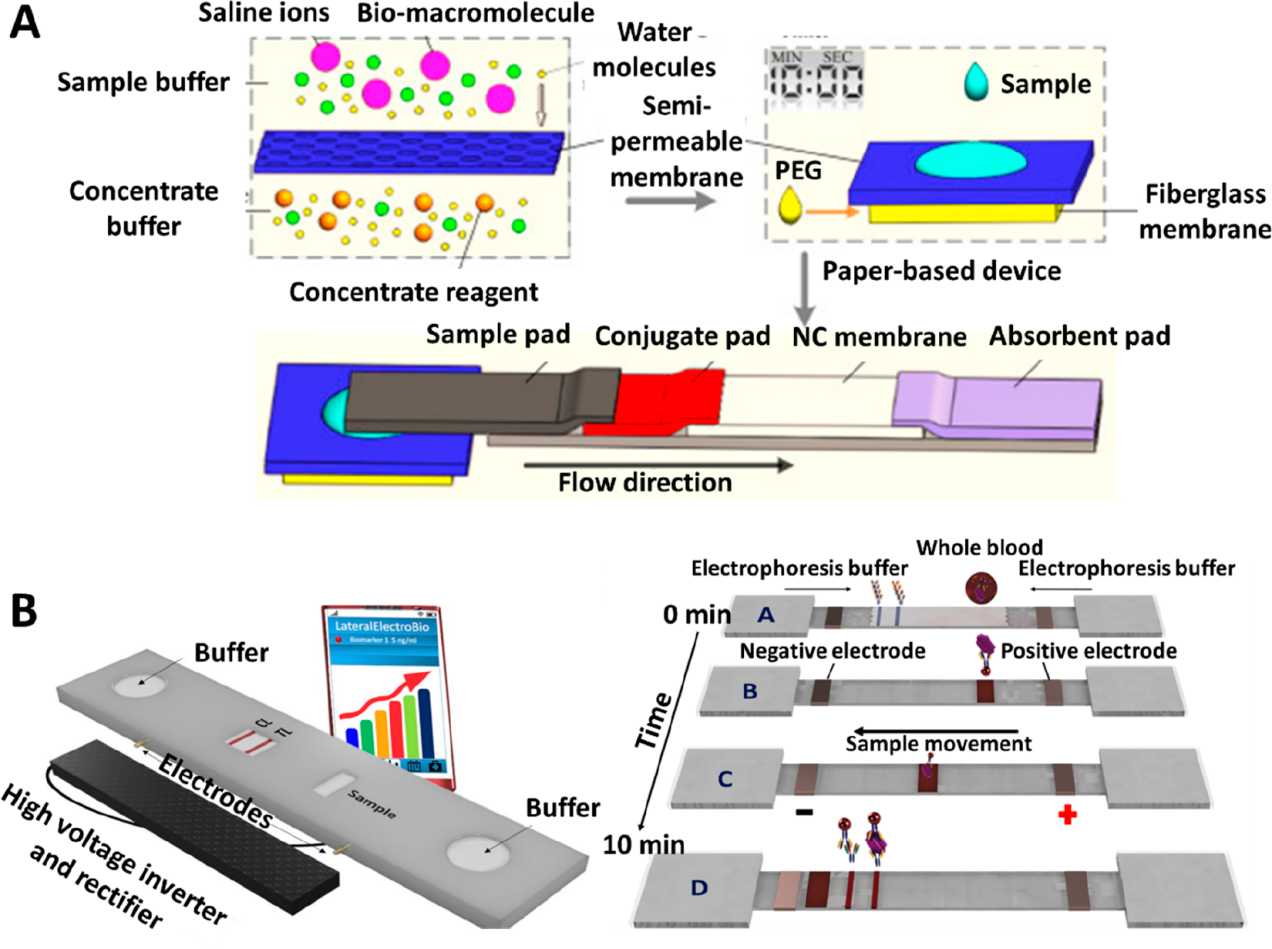
(A) Schematic representation of a 10 min preconcentration approach based on the incorporation of a dialysis membrane onto the sample pad of an LFA strip. PEG conjugated to a fiberglass membrane is used as the dialysate. Reprinted (adapted) with permission from ref (173). Copyright 2016 Elsevier. (B) Schematic representation of a paper electrophoretic device powered by a smartphone battery. The whole blood sample is driven by electrophoresis toward the detection zone, avoiding the clogging on the nitrocellulose pores. Reprinted (adapted) with permission from ref (71). Copyright 2021 American Chemical Society.

Sample preconcentration can also be performed by the integration of physical separation techniques in LFA. For example, Tang et al. have integrated dialysis into an LFA for the preconcentration of the sample. They incorporate a semipermeable membrane (MWCO 3.5 KD) onto a fiberglass sample pad that has been pretreated with PEG. The highly hygroscopic nature of the PEGylated fiberglass allows it to act as a dialysate in this system (Figure 13A). With this approach, the authors report that a 10 min preconcentration resulted in a 10-fold and 4-fold signal enhancement in the detection of HIV specific nucleic acid sequences and myoglobin, respectively. This strategy shows great promise for PoC applications given its simplicity, speed, and low cost; however, the approach still needs to be validated with real-world samples.^173^

Moghadam et al. have used isotachophoresis in LFAs for integrated sample extraction and preconcentration. Isotachophoresis is a nonlinear, equipment-free, electrophoretic technique where leading (LE) and trailing (TE) electrolytes are used to focus the analyte in a reduced area. This technique was found to improve the binding affinity between human IgG (HIgG) and anti-HIgG resulting in a 1 order of magnitude increase in the concentration of analyte bound at the TL and a 400-fold improvement in the LoD of the assay, when compared to the conventional LFA. Additionally, the authors show that isotachophoresis improved analyte extraction from the sample matrix; in their case, up to 80% of IgG could be extracted from real human serum samples.^174,175^

On a similar note, Sena et al. have developed a portable and low-cost paper-based electrophoretic bioassay (PEB) that enables the detection of HIgG in neat whole blood samples. A potential of 200 V was applied across the nitrocellulose test strip using a smartphone in combination with a joule thief inverter and rectifier, which is compatible with the use of this assay in PoC scenarios where resources are limited. The electrophoresis drives the whole blood sample to the detection zone, avoiding the clogging of the nitrocellulose pores. Any excess blood can then be electrophoretically driven off of the detection zone allowing a clear visual readout that can be easily quantified using a smartphone for image capture and analysis (Figure 13B). This approach allowed for the assay sensitivity in whole blood to reach the same levels of those obtained in the lab with PBS. These methodologies are new and require further optimization and analysis but clearly have the potential for use in future LFAs in complex media.

### 5.3 Bioreceptor Engineering

A more sophisticated way to improve the analytical capability of LFA when evaluating complex samples is to improve the analytical properties of the bioreceptors. Traditional bioreceptors include DNA fragments and antibodies, but in recent years, novel bioreceptors such as aptamers and antibody fragments have garnered much interest.

Aptamers are nucleic acid sequences (normally DNA or RNA) that fold into three-dimensional conformations that allow them to specifically bind to analytes of interest.^176^ Aptamer selection is performed through a controlled *in vitro* process (using the Sequential Evolution of Ligands by Exponential Enrichment, SELEX) that allows not only for nonselective sequences to be discarded but also for those sequences that do bind to the analyte of interest to be ranked and selected based on their binding affinity.^177^ While the binding constants tend to be similar to those of antibodies, aptamers generally produce a more specific response. There are three main reasons for this: (i) They have lower molecular weights than antibodies, decreasing the chances of nonspecific interactions. (ii) Being nucleic acids, their interaction with other bioreceptors is lower. (iii) During the SELEX process, it is possible to include a counter-selection step, which actively removes all aptamer sequences that nonspecifically bind to possible contaminants. Huan et al. have recently reviewed the application of aptamers in LFAs, focusing on the combination of these bioreceptors with signal amplification and multiplexing strategies.^178^

Similar to aptamers, antibody fragments and nanobodies can also improve the specificity of LFA when employing complex samples. Antibody fragments are generated by the proteolytic enzymatic fragmentation of full size antibodies (mostly IgG). In this way it is possible to remove the Fc region of IgG, which is usually responsible of nonspecific interactions with endogenous Fc receptors. Additionally, the presence of less charged groups reduces the possibility of cross-reactivity.^19^ However, to the best of our knowledge there are still no reports proving the aforementioned analytical capabilities of antibody fragments when evaluating complex samples in LFA. On the contrary, nanobodies (or single domain antibodies, sdAb) are the single chain binding portions of camelid antibodies. Thanks to their low molecular weight (<15 kDa), easy fabrication (being a single chain of amino acid, they can be produced by *E. coliin vitro*), and stability, they seem like an ideal bioreceptor for LFA. In this context, few works have employed them to enhance the LFA capabilities. Between them, the work from Loynachan et al. combined nanobodies with platinum nanocatalysts to achieve the naked-eye detection of p24 spiked into sera in the low femtomolar range, as well as the detection of acute-phase HIV in clinical human plasma samples.^179^

The implementation of the CRISPR/Cas recognition systems in LFAs has been reported to provide both high specificity and sensitivity.^180^ This method has recently been utilized for the dual detection of the envelope (E) and open reading frame 1ab (Orf1ab) genes of SARS-CoV-2 in nasopharyngeal swab samples by Xiong et al. The use of CRISPR/Cas9 with multiplexed RT-RPA enabled the amplification of the target genes without the need for external instrumentation. Cas9/sgRNA complex formation eliminates interference from the residual primer-dimers, improving the assay specificity. The authors report a high clinical sensitivity (97%) and specificity (100%) after evaluating 64 real patient samples.^181^

**Figure 14:**
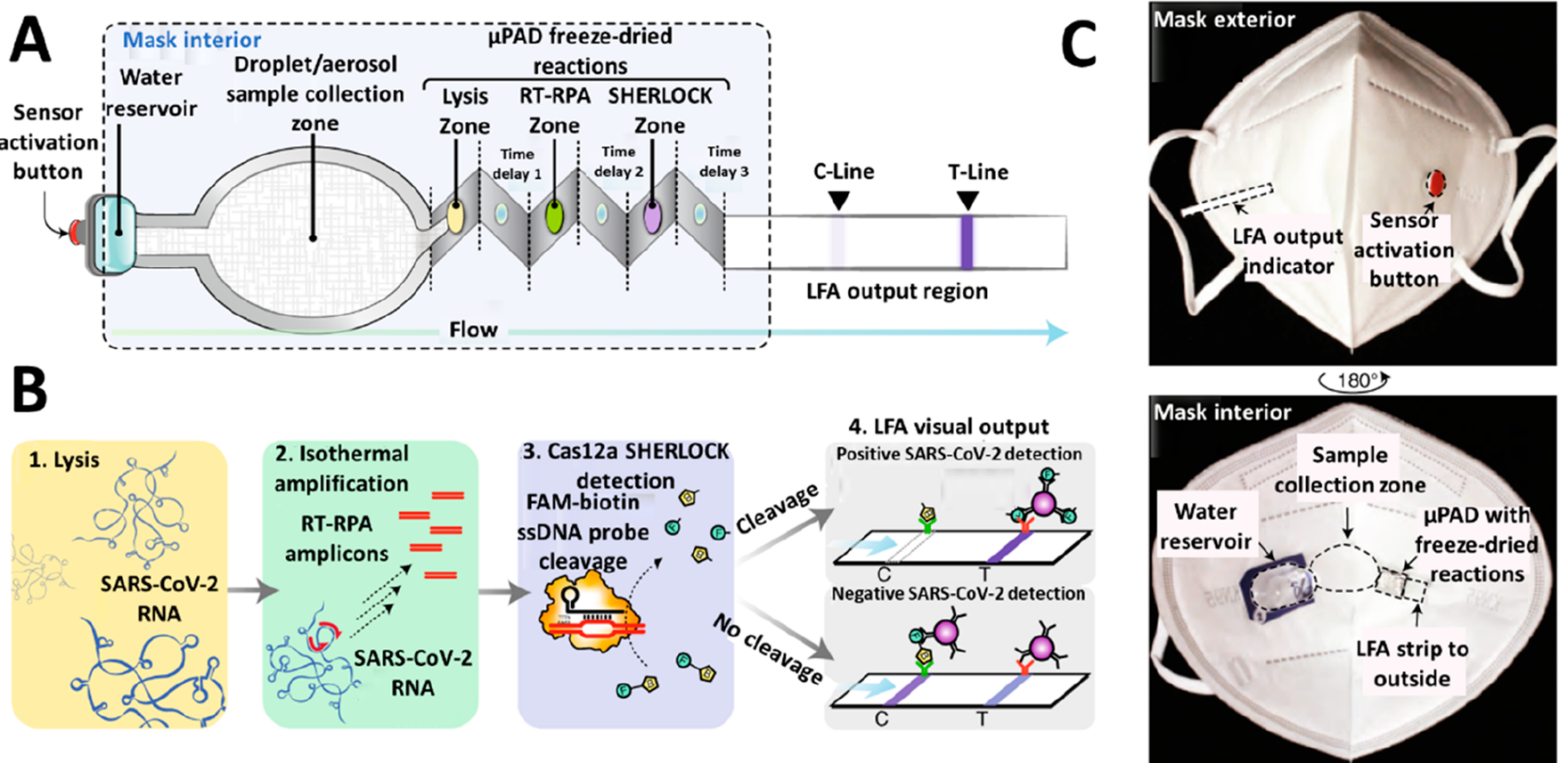
(A) Schematic representation of the face mask-LFA capable of detecting exhaled SARS-CoV-2. Once the water reservoir is pressed, the water flow moves the collected aerosol sample toward the paper μPAD, which contains the freeze-dried assay reagents. (B) First, the RNA is extracted, and then, it is amplified by RT-RPA and detected by the Cas12a SHERLOCK method. The output is visualized in an LFA strip that is built into the face mask. (C) Pictures of the face mask interior and exterior. Reprinted (adapted) with permission from ref (182). Copyright 2021 Springer Nature.

Nguyen et al. have developed an interesting platform that utilizes isothermal amplification and Cas12a SHERLOCK for the detection of exhaled SARS-COV-2 on a face mask. The aerosol collection and nucleic acid amplification are performed on the interior surface of the face mask, while the detection step is done by the integration of a dedicated LFA (Figure 14). This approach showed high selectivity over other human coronavirus strains and had an LoD of 500 RNA copies.^182^

## 6 LFA Validation and Barriers toward Commercialization

The LFA market has seen a fascinating growth in recent decades, from USD 2.2 billion in 2005 to USD 8.2 billion in 2020, which has been driven by the high prevalence of infectious diseases and the increasing popularity of home-diagnosis.^183^ The outbreak of the COVID-19 pandemic caused a boom in the market by rapidly increasing the demand for at-home (PoC) test kits.^184^ Despite their lower accuracy compared to RT-qPCR, LFAs have still proven popular due to their point-of-care properties. Given this, the LFA market size is expected to reach USD 10.2 billion by 2025.^185^

As with any technology, one method of estimating how close a particular LFA is to market is by assessing its TRL level. This is a system used to assess the maturity of a particular technology.^137^ For the different stages of LFA development,

1. TRL-1 would correspond to the achievement of a measurable signal in the TL upon the detection of a target analyte using a half-stick format;
2. TRL-2 to the identification of the ideal components to build the full LFA;
3. TRL-3 to the determination of the LFA properties (sensitivity, specificity, dynamic range, RSD, etc.) after the evaluation of serial dilutions of the analyte in buffer in the laboratory;
4. TRL-4 to the determination of the LFA accuracy when detecting the analyte in real samples (prevalidated using gold standard methods) in the laboratory;
5. TRL-5 to the validation of the LFA with prevalidated real samples in the hospital with trained users;
6. TRL-6 to the validation of the LFA with clinical samples in the hospital with trained personnel;
7. TRL-7 to the validation of the LFA in the hospital using clinical samples and untrained personnel;
8. TRL-8 to the assessment of the commercial LFA in deployment conditions, proving full compliance of the quality standards and regulations; and
9. TRL-9 to the achievement of a fully operational LFA, ready for commercialization, with the system optimized for full rate production.

Most of the technology (with a few exceptions) discussed in sections 2–5 of this Review are at TRL levels 1–5. Commercially available LFAs are relatively simple devices not utilizing the technological advancements being reported in research laboratories. This begs the question of what barriers are preventing the commercial adoption of these technological advances?

One possible reason is that research centers and small companies suffer a funding gap between TRLs 4 and 6, also known as the “valley of death”.^186^ This is the point in assay development when tests move away from in-the-lab measurements with ideal conditions and simulated samples (TRL 4) to validation with real-world samples at the intended point of use (TRLs 5 and 6). Most investors only become interested when technologies reach TRLs 7–9.^187^ In an attempt to correct this issue, funding organizations (e.g., the European Commission) and governments have started to increase funding to collaborative projects between academia and industry.^188−190^ The involvement of industrial partners throughout the entire LFA development process is intended to encourage stricter critical evaluation of LFA cost, performance, manufacturing capability, and scalability, properties that are often overlooked in academic research. In addition to this, the involvement of industrial partners as early as possible is intended to speed up the time to market of LFAs.^191−193^

Another important barrier toward commercialization is to obtain all the regulatory approval required for product launch. This is a tedious and expensive process that starts with the analytical and clinical validation of the test. The analytical validation assesses the precision, accuracy, and reliability of the test, while the clinical validation evaluates the parameters such as the diagnostic sensitivity, specificity, positive and negative predictive value, and likelihood ratio of the LFA.^194^ There are several companies offering support services to diagnostic companies during this process.^195,196^

For goods intended to be sold in the European Union (EU), a CE mark is required. In order to obtain this certification, LFA manufacturers must submit a performance evaluation report to the regulatory agency showing the analytical and clinical validation of their LFA. The *in vitro* diagnostic medical devices regulation (IVDR) (EU, 2017/746) classifies devices in relation to their risk if inaccurate; in other words, if the test gives a false negative, what risk is posed to the general population. Tests are assigned a letter of the alphabet (A, B, C, or D) based on their score in this test. Diagnostics for the detection of pregnancy, cholesterol level, glucose, or bacteria in urine are classified as moderate health risks (B), while assays for transmissible agents with a high risk of propagation (e.g., SARS-CoV-2) are classified as D. The next step is a conformity assessment, in which an independent certified body evaluates the analytical and clinical performance of the test.^197^

In America, the Food and Drug Administration (FDA) has a similar regulation, where the tests are classified as I, II, or III depending on their health risk in case of inaccuracy. Most LFAs are class II and must undergo a stringent premarket review (only class I is exempt), where their analytical and clinical performance is evaluated and compared with equivalent tests or with a standardized reference method.^198^

The European and American regulators are the strictest and hardest to obtain. If a test is developed that passes these regulations, then they will normally be accepted worldwide.

The main obstacles to commercialization are therefore not necessarily scientific issues, but rather difficulties in raising capital for low TRL level technologies and the high costs (both financial and in terms of time) and complexity of obtaining regulatory approval.

## 7 Outlook

In this paper, we have reviewed the advances in LFA from the last ten years with an emphasis on the role of nanomaterials. We have identified four main areas that should simultaneously be improved to achieve the next generation of LFA: sensitivity enhancement, multiplexing, quantification at the PoC, and evaluation of complex samples. These improvements need to retain the key advantageous properties of LFAs, namely, their low cost, rapid time to result, and ease of operation interpretation.

Notable advances have been made that increase the sensitivity of LFAs. These are important as assays are being developed for low-concentration analytes, increasing the utility of LFAs. Sensitivity can be enhanced using novel reporters, signal amplifiers, and alternative signal transduction methods. Likewise, advances in multiplexing methodologies have been reviewed and are sure to form a large part of future research and commercial LFA products. Technologies that allow the detection of multiple analytes in a single TL are perhaps the most interesting as they allow the exponential increase in the number of analytes that can be detected in an LFA, since they can be combined with novel device designs that allow multiple target analytes to be investigated in a single test. Likewise, microarrays optimize the available test area for the detection of a multitude of analytes in a single detection zone.

While there has been a miniaturization of LFA readers in recent years to analyze and quantify the signal on test strips, the past decade has seen a real trend in the use of smartphones for LFA analysis. This often requires portable boxes or cassettes to control the lighting conditions of image acquisition with a smartphone camera. The advent of portable 3D printers is allowing the fabrication of many of these at the point of use. In the future the increasing power of smartphones and the advent of 5G networking promise to further improve PoC LFAs, in terms of both their analytical performance and their data analysis. The development of point-of-care readers that enable PoC analysis using alternative detection methods (such as SERS, TCA, and MPQ) is still in its infancy but undoubtedly has potential. We anticipate that the next decade will see an increase in research to reduce the cost and improve the performance of such readers for LFA applications.

The simplicity of use of LFAs is what makes them so desirable; it is therefore encouraging to see the work by researchers to incorporate sample pretreatment methods into LFAs. Any technological developments aimed at allowing the analysis of untreated sample matrices is of great interest to the entire field of LFA development. The future of LFAs is undoubtedly a “closed box” system for the end user where they add their sample and get a simple, reliable, and quantified readout. Such devices will most likely include combinations of the technologies reviewed herein. The practice of combining technologies is rare, with most publications understandably focusing on a single, novel technological advancement. The combination of several technological advancements in a single LFA has the potential to make a greater societal impact and should be given encouragement.

Throughout this Review, we have seen that there are various techniques that have been employed to address the four main challenges in the field: quantification at the PoC, sensitivity enhancement, multiplexing, and the evaluation of complex samples. While all of the techniques investigated require more development, it is worth considering which are currently the most promising. This is a largely subjective exercise, but the authors would suggest that, of all the techniques covered, perhaps the use of SERS in LFAs is the most advanced and currently the best able to solve the four main challenges highlighted in this Review. We have seen that portable SERS readers are being successfully developed for on-site quantification of signals in LFAs;^59^ there are improved sensitivities when using SERS compared to colorimetric detection, with LoDs as low as pg/mL being reported.^123^ Different SERS nanotags that emit different SERS signals have been employed for multiplexing,^136^ and while SERS is naturally more suited to complex media analysis than colorimetric or fluorescent measurements, the ability to couple SERS labels to magnetic nanoparticles has also facilitated complex media analysis.^103^ The greatest challenges in SERS-based LFA development concern the readers, which need much more development to be practically usable. Current limitations include high costs, long data acquisition times, and not being very user-friendly. The authors believe work should be conducted to increase the scan speed and software developed to give simple-to-understand readouts. Unlike optical readout methods, the results of SERS are not intuitively understood, so software that can accurately and reliably process, interpret, and report the results in an easy-to-understand fashion is required for real-world applications where the assays will be performed by untrained personnel.

Advances in the other technologies discussed in this Review (such as TCA) show that they are very promising and developing well; however, at the time of writing, they are not able to solve all four of the issues discussed (multiplexing with TCA has not been demonstrated yet). Further development of these techniques to address these issues is required, before integrating those developments into a single sensing model, which means they are some years behind SERS. Similarly, the recently published work by Miller et al. also achieves quantification and ultralow sensitivity;^84^ however, it would probably require extensive sample treatment, and its ability to detect multiple targets would require the printing of multiple detection zones.

There is an obvious trend in the literature to develop new methods of signal transduction. There have been decades of research and development of optical (colorimetric and fluorescent) LFAs, and their limitations seem to have been reached. They lack the sensitivity and reliability to detect the biomarkers of interest for future sensing applications, currently only being able to detect highly concentrated biomarkers. Different types of energy (sound and thermal) and forces (magnetism) are being used to replace light in LFAs; we envisage that in the future more development of these and the use of other signal transduction methods will be prominent in LFA development.

## List of Abbreviations

AgNBA@Au: silver core and gold shell NPs loaded with Nile blue A dye
AgNPs: silver nanoparticles
AFB1: aflatoxin B1
ALP: alkaline phosphatase
AuNPs: gold nanoparticles
BoNT: botulinum neurotoxin
CAS: cellular apoptosis susceptibility
CCD: charge-coupled device
CdSe@ZnS QDs: cadmium selenide/zinc sulfide quantum dots
CdTe QDs: cadmium telluride quantum dots
CFU: colony-forming unit
CK-MB: creatine kinase myocardial band
CL: control line
CMOS: complementary metal-oxide-semiconductor
CPU: central processing unit
CrAg: cryptococcal antigen
CRISPR: clustered regularly interspaced short palindromic repeats
cTnI: cardiac troponin I
*C. difficile*: *Clostridium difficile*
DENV: dengue virus
DNA: deoxyribonucleic acid
DTNB: 5,5′-dithiobis(2-nitrobenzoic acid)
*E. coli*: *Escherichia coli*
ELISA: enzyme-linked immunosorbent assay
Ems.: emission
et al.: et alia (and others)
Exc: excitation
EVD: Ebola virus disease
FAM: carboxyfluorescein
FDA: Food and Drug Administration
GDH: glutamate dehydrogenase
GO: graphene oxide
GPS: global positioning system
HAdV: human adenovirus
HAV: hepatitis A virus
hCG: human chorionic gonadotropin
HCV: hepatitis C virus
HIgG: human immunoglobulin G
HIV: human immunodeficiency virus
HPV: human papillomavirus
HRP: horseradish peroxidase
H1N1: subtype of the type A influenza virus
IgG: immunoglobulin G
IgM: immunoglobulin M
ITP: isotachophoresis
IU: international units
IVD: in vitro diagnostics
IVDR: in vitro diagnostics regulations
KD: thermodynamic equilibrium dissociation constant
kDa: kilo-Dalton
LATE: linear-after-the-exponential
LDW: laser direct writing
LE: leading electrolyte
LED: light-emitting diode
LFA: lateral flow assay
LoD: limit of detection
LoQ: limit of quantification
LSPR: localized surface plasmon resonance
Meth: methamphetamine
miRNA: micro-RNA
MPQ: magnetic particle quantification
MWCNTs: multiwalled carbon nanotubes
MWCO: molecular weight cut-off
Myo: myoglobin
NIR: near-infrared radiation
NPs: nanoparticles
N,S-doped: nitrogen- and sulfur-doped
Orf1ab: open reading frame 1ab
PA: photoacoustic
PBS: phosphate buffer saline
PCL: polycaprolactone
PCR: polymerase chain reaction
PEB: paper-based electrophoretic bioassay
PEG: polyethylene glycol
PES: polyester
pH: potential/power of hydrogen
PoC: point-of-care
PSA: prostate specific antigen
Pt: platinum
PtNCs: platinum core–shell nanocatalysts
QDs: quantum dots
RBP: retinol-binding protein
RGB: red green blue
RNA: ribonucleic acid
ROX: carboxyrhodamine
RPA: recombinase polymerase amplification
rpm: revolutions per minute
R&D: research and development
SARS-CoV-2: Severe Acute Respiratory Syndrome Coronavirus 2
SEM: scanning electron microscopy
SERS: surface-enhanced raman scattering
sgRNA: single guide RNA
SHERLOCK: specific high-sensitivity enzymatic reporter unlocking
SNPs: single nucleotide polymorphisms
SiO2@DQs: silica-core@dual quantum dot (QD)–shell nanotags
SPIONS: superparamagnetic iron oxide nanoparticles
SPR: surface plasmon resonance
ssDNA: single-stranded DNA
ST2: soluble interleukin 1 receptor-like 1
TCA: thermal contrast amplification
TE: trailing electrolyte
TEM: transmission electron microscopy
TL: test line
TMB: 3,3′,5,5′-tetramethylbenzidine
TRL: technology readiness level
TSH: thyroid-stimulating hormone
UCNPs: upconverting nanoparticles
USD: United States dollar
UV: ultraviolet
WHO: World Health Organization
YFV: yellow fever virus
ZEBOV: Zaire Ebola virus
ZrMOFs: zirconium metal organic frameworks
3D: 3-dimensional
